# Self‐Assembly of Antigenic Peptide Nanofibrils Templates the Growth of Silica Nanoparticles for Nanovaccines

**DOI:** 10.1002/advs.76636

**Published:** 2026-07-14

**Authors:** Xuecheng Yang, Min Li, Zhiying Yao, Yu Hu, Zhili Wang, Wendi Shi, Bingbing Sun

**Affiliations:** ^1^ School of Chemical Engineering Dalian University of Technology Dalian China; ^2^ State Key Laboratory of Fine Chemicals School of Chemical Engineering Dalian University of Technology Dalian China; ^3^ Frontiers Science Center for Smart Materials Oriented Chemical Engineering Dalian University of Technology Dalian China

**Keywords:** Alzheimer's disease, human papillomavirus, peptide vaccines, self‐assembly, silica, vaccine adjuvants

## Abstract

The low immunogenicity of peptide vaccines remains a critical challenge in immunotherapy. While peptide nanofibrils can function as self‐delivery systems to activate antigen‐presenting cells (APCs), most existing designs rely on complex covalent conjugation to β‐sheet‐forming motifs. Moreover, the diverse surface properties of various antigenic peptides complicate the rational selection of appropriate adjuvants. Herein, we develop a nanovaccine platform in which antigenic peptides self‐assemble into nanofibrils without the need for exogenous β‐sheet‐forming sequences. Silica nanoparticles (SiO_2_ NPs) are nucleated and grown directly along these fibrils, resulting in SiO_2_@fibril nanovaccines with a unique “beads‐on‐a‐string” morphology. Using amyloid‐β(1‐42) (Aβ42) and human papillomavirus (HPV) type 16 E7 (E7) as model antigens, we demonstrate that these nanovaccines significantly enhance the maturation and activation of bone marrow‐derived dendritic cells (BMDCs). In an Alzheimer's disease animal model using APP/PS1 mice, SiO_2_@Aβ42 nanovaccines improve motor and cognitive function. Additionally, in an HPV animal model using TC‐1 tumor‐bearing mice, SiO_2_@E7 nanovaccines suppress tumor growth and increase survival rate. This strategy provides a universal and modular adjuvant platform for peptide‐based nanovaccines.

## Graphical abstract

Antigenic peptides Aβ42 and E7 self‐assemble into nanofibrils; APTES and TEOS induce SiO_2_ NP formation on these fibrils to form SiO_2_@fibril nanovaccines, which activate BMDCs in vitro. In vivo, SiO_2_@Aβ42 fibril nanovaccines alleviate AD symptoms and clear Aβ42 plaques in APP/PS1 mice, and SiO_2_@E7 fibril nanovaccines inhibit tumor growth and promote survival rate in TC‐1 tumor‐bearing mice.

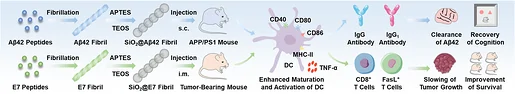

## 1 Introduction

Peptide vaccines have been widely used in immunotherapy because of their high safety, biocompatibility, specificity, and structural design flexibility [1, 2], and have been applied in the treatment of neurodegenerative diseases, cancers, infectious diseases, and autoimmune diseases [3, 4, 5, 6]. However, as small molecules, antigenic peptides exhibit poor stability after injection, insufficient maturation and activation of antigen‐presenting cells (APCs), a deficient immune response, and weak immune memory, resulting in an ineffective immune response [7, 8, 9, 10]. The ineffective immune response limits the clinical translation and therapeutic efficacy of peptide‐based vaccines, so improving the efficiency of the immune response has become the key issue and major challenge for peptide‐based vaccines.

Numerous strategies are employed to enhance the immune responses of antigenic peptides, such as optimization of peptide sequence [11, 12] and rational design of appropriate delivery systems [13, 14]. Among these strategies, self‐assembling antigenic peptides into nanostructures, e.g., nanofibers and nanospheres, constitute a self‐delivery system and have been widely used to enhance the immunostimulatory capacity of antigenic peptides [15, 16]. For example, Covalent modification of antigenic peptides with β‐sheet‐forming sequences enables the self‐assembly of the modified peptides into nanofibrils rich in β‐sheet. This self‐assembly provides a self‐delivery platform that enhances specific immune responses by promoting the uptake of antigenic peptides by APCs and the maturation and activation of APCs [17, 18, 19, 20]. OVA_323‐339_, an antigenic peptide comprising 17 amino acids, can self‐assemble into nanofibers after covalent conjugation with β‐sheet‐forming sequences such as Q11 or KFE8. Compared with unassembled antigenic peptides, these nanofibers induce higher levels of activation and maturation of APCs, antibody responses, and T cell‐dependent humoral immune responses [17, 18, 19]. Similarly, the E7_44‐62_ peptide derived from human papillomavirus type 16 (HPV16) E7 protein can also self‐assemble into nanofibers after covalent conjugation with the Q11 sequence. The self‐assembled nanofibers elicit enhanced cellular immunity and CTL responses, as well as improved anti‐tumor efficacy [20]. However, the formation of nanofibrils in current studies is dependent on covalent coupling between antigenic peptides and β‐sheet‐forming sequences. The introduction of the β‐sheet‐forming sequences may disrupt the presentation of antigenic epitopes, further impairing the specificity and effectiveness of the immune responses [21, 22]. Additionally, because different antigenic peptides exhibit distinct surface properties, e.g., surface charges and hydrophilic‐hydrophobic properties, the selection of universal delivery systems and vaccine adjuvants for this peptide vaccine strategy is challenging [23, 24].

To address these issues, we designed a novel nanovaccine platform in which antigenic peptides self‐assembled into nanofibrils without the introduction of β‐sheet‐forming sequences, and then silica nanoparticles (SiO_2_ NPs) formed through nucleation and growth along the antigenic nanofibrils. This strategy produced a unique morphology of SiO_2_ NPs coating antigenic peptide fibrils, named SiO_2_@fibril nanovaccines. We selected antigenic peptides amyloid‐β(1‐42) (Aβ42) and Human Papillomavirus type 16 E7 (E7) as model antigens to evaluate the immunostimulatory capacity of SiO_2_@fibril nanovaccines. Our results demonstrated that SiO_2_@fibril nanovaccines served as a universal vaccine platform that more effectively stimulated the maturation and activation of bone marrow‐derived dendritic cells (BMDCs) and induced stronger antigen‐specific immune responses than antigenic nanofibrils. The immunotherapeutic effects of SiO_2_@Aβ42 fibril nanovaccines and SiO_2_@E7 fibril nanovaccines were validated in specific animal models, including APP/PS1 mouse model and TC‐1 tumor‐bearing mouse model, respectively. In summary, the design of SiO_2_@fibril nanovaccines not only ensured the integrity of the antigenic epitopes but also provided a universal antigen‐delivery strategy.

## 2 Results

### 2.1 Preparation and Characterization of the SiO_2_@Aβ42 Fibril Nanovaccines

The SiO_2_@Aβ42 fibril nanovaccines were prepared using a method in which SiO_2_ nanoparticles (NPs) nucleated and grew along the antigen peptide fibrils (Figure 1A). First, Aβ42 monomers were incubated to form mature nanofibrils (Figure 1B and Figure S2). Thioflavin T (ThT) kinetic analysis demonstrated that Aβ42 fibrillation involves the accumulation of β‐sheet secondary structure (Figure S1). Subsequently, (3‐aminopropyl) triethoxysilane (APTES) and tetraethyl orthosilicate (TEOS) were added to the Aβ42 nanofibrils to drive the nucleation and growth of SiO_2_ NPs along their surface. APTES adsorbed onto the Aβ42 nanofibrils and served as SiO_2_ nucleation sites, while TEOS facilitated the continuous growth of SiO_2_ NPs along these APTES nuclei. This resulted in the formation of SiO_2_ NPs‐coated Aβ42 fibril nanovaccines (SiO_2_@Aβ42 fibril nanovaccines) (Figure 1B and Figure S2). Dynamic light scattering (DLS) analysis indicated that the hydrodynamic sizes of the Aβ42 fibrils and SiO_2_@Aβ42 fibril nanovaccines were 530 ± 246 and 1330 ± 135 nm, respectively. Although the inherent limitations of DLS in measuring high‐aspect‐ratio materials, it remained viable for determining their hydrodynamic sizes [25]. Their zeta potentials were ‐34±1 and 33±1 mV, respectively (Table S1). The reverse of the zeta potential of Aβ42 fibrils from negative to positive after SiO_2_ formation on their surface indicated that the SiO_2_@ Aβ42 fibril nanovaccines were coated with a positively charged SiO_2_ layer containing exposed amine groups from APTES [26]. Transmission Electron Microscope (TEM) size measurements indicated that the distribution of their widths was uniform, with widths of 10 ± 2 nm and 23 ± 3 nm, respectively (Table S1 and Figure 1C). These results indicated that the SiO_2_@ Aβ42 fibril nanovaccines prepared using a nucleation‐growth strategy for SiO_2_ NPs along Aβ42 fibrils exhibited a uniform size distribution, and their diameter was nearly doubled after SiO_2_ NPs formation on the surfaces of Aβ42 fibrils. Energy‐dispersive X‐ray spectroscopy (EDS) mapping demonstrated distinct colocalization of Si and O elements in the SiO_2_@Aβ42 fibril nanovaccines (Figure 1D), indicating the formation of SiO_2_ NPs along the surface of Aβ42 fibrils. Inductively coupled plasma‐optical emission spectrometry (ICP‐OES) analysis further quantified the mass ratio of Si to Aβ42 in the SiO_2_@Aβ42 fibril nanovaccines as 3.07 ± 0.55 (Figure 1E). The thermal stability of Aβ42 fibrils and SiO_2_@Aβ42 fibril nanovaccines was analyzed using differential scanning calorimetry (DSC). DSC analysis showed that Aβ42 fibrils exhibited sustained exothermic behavior during continuous heating from 20°C to 100°C (Figure S3A), indicating that Aβ42 fibrils underwent a sustained heat‐induced aggregation [27]. SiO_2_@Aβ42 fibril nanovaccines exhibited a transition temperature (T_m_) of 54.76°C ± 0.17°C (Figure S3B), indicating that SiO_2_@Aβ42 fibril nanovaccines remained thermally stable below the T_m_.

**FIGURE 1:**
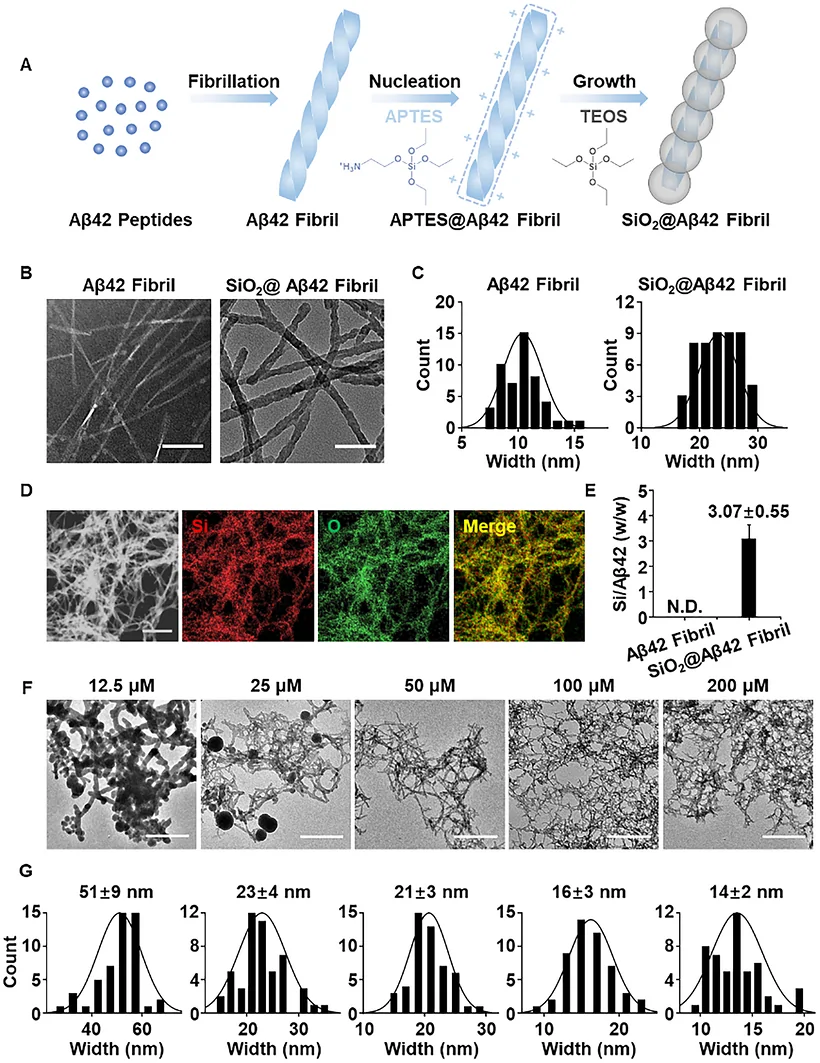
Preparation and characterization of the SiO_2_@Aβ42 fibril nanovaccines. (A) Formation mechanism of the SiO_2_@Aβ42 fibril nanovaccines. (B) TEM images of the Aβ42 fibrils and SiO_2_@Aβ42 fibril nanovaccines. The scale bar is 100 nm. (C) Width distributions of the Aβ42 fibrils and SiO_2_@Aβ42 fibril nanovaccines. (D) EDS analysis of the co‐localization of Si and O in the SiO_2_@Aβ42 fibril nanovaccines. The scale bar is 500 nm. (E) ICP‐OES analysis of the mass ratios of Si to Aβ42 in the Aβ42 fibrils and SiO_2_@Aβ42 fibril nanovaccines. *n* = 3. (F) TEM images showing the effects of Aβ42 concentrations on the morphologies of the SiO_2_@Aβ42 fibril nanovaccines. The scale bar is 500 nm. (G) Width distributions of the nanovaccines prepared at different Aβ42 concentrations.

To explore the detailed growth mechanism of the SiO_2_@Aβ42 fibril nanovaccines, the effect of the concentration of Aβ42 peptide on the formation of SiO_2_@Aβ42 fibril nanovaccines was investigated. During the formation of SiO_2_@Aβ42 fibril nanovaccines, a lower concentration of Aβ42 peptide resulted in wider NPs, accompanied by the formation of free spherical SiO_2_ NPs. A higher concentration of Aβ42 peptide resulted in narrower, incompletely nucleated NPs (Figure 1F). In detail, when the concentration of Aβ42 was 12.5 µm, the width of the nanovaccines was 51 ± 9 nm, accompanied by the generation of free spherical SiO_2_ NPs. As the concentrations of Aβ42 increased to 25, 50, 100, and 200 µm, the widths of the nanovaccines decreased to 23 ± 4, 21 ± 3, 16 ± 3, and 14 ± 2 nm, respectively, and the free spherical SiO_2_ NPs gradually disappeared (Figure 1G). This result indicated that Aβ42 fibrils served as a nucleation‐growth template for SiO_2_ NPs and modulated SiO_2_ deposition on their surfaces. To confirm the structural integrity of the coated antigenic peptides after release, an enzyme‐linked immunosorbent assay (ELISA) was conducted. The results of the ELISA confirmed that the silica‐released antigen was recognized by antibodies to a similar extent as the unencapsulated antigen (Figure S4), thereby demonstrating the preservation of intact epitopes of the antigenic peptides.

A secondary nucleation‐growth strategy in which SiO_2_ NPs were grown along the primary product was employed to prepare secondary SiO_2_@Aβ42 fibril nanovaccines to increase the mass ratio of Si to Aβ42. The SiO_2_@Aβ42 fibril nanovaccines prepared by the secondary nucleation‐growth of SiO_2_ NPs still retained the morphology of SiO_2_ NPs‐coated Aβ42 fibril (Figure S6A). The mass ratio of Si to Aβ42 increased to 9.69 ± 1.34 (Figure S6B), indicating a significant increase in SiO_2_ content in the secondary products compared with the primary products. The widths of secondary products increased to 42 ± 6 nm (Table S1), approximately twice those of the primary products. The hydrodynamic size of secondary products was 3728 ± 162 nm (Table S1). The increases in the hydrodynamic size of the secondary products corresponded to the increased widths compared with the primary products. The zeta potential of secondary products was −33 ± 1 mV (Table S1). It was interesting to note that the zeta potential of secondary products shifted from positive to negative. This change may be attributed to incomplete growth of TEOS along the APTES nuclei in the primary products, resulting in numerous positively charged APTES nuclei still exposed on their surfaces. In the secondary products, TEOS grew more completely and covered these nuclei in the secondary products, leading to a negative zeta potential [28].

Additionally, an Aβ40 fibril was selected as another template for the nucleation‐growth of SiO_2_ NPs. It was shown that uniform SiO_2_@ Aβ40 fibril NPs were formed at an Aβ40 peptide concentration of 100 µm. Lower concentrations of Aβ40 resulted in wider NPs accompanied by the formation of spherical SiO_2_ NPs, whereas higher concentrations of Aβ40 resulted in narrower, incompletely nucleated NPs (Figure S5). This result suggested that this is a universal platform for the formation of SiO_2_@ fibril NPs.

### 2.2 SiO_2_@Aβ42 Fibril Nanovaccine‐Induced Bone Marrow‐Derived Dendritic Cells (BMDCs) Maturation and Activation

Due to the increased amount of SiO_2_ on the secondary product for sufficient activation of immune responses, the secondary SiO_2_@Aβ42 fibril nanovaccines were used in the following in vitro and in vivo studies. Dendritic cells (DCs) are prototypical antigen‐presenting cells (APCs) and their maturation and activation indicate efficient antigen uptake, processing, and presentation [29]. Therefore, bone marrow‐derived dendritic cells (BMDCs) were selected as a cellular model. The expression of surface markers on BMDCs and the secretion of cytokines by BMDCs are key indicators of their activation and maturation [30, 31, 32]. The secondary SiO_2_@Aβ42 fibril nanovaccines exhibited excellent biocompatibility and showed negligible cytotoxicity toward BMDCs in the range of 0–100 µg/mL (Figure S7). The SiO_2_@Aβ42 fibril nanovaccines induced the maturation and activation of BMDCs. The nanovaccines induced CD11c^+^ cells to express surface markers CD40, CD80, CD86, and MHC‐II at rates of 37.20% ± 1.93%, 54.60% ± 0.67%, 37.77% ± 2.02%, and 52.80% ± 2.61%, respectively, significantly higher than those observed with Aβ42 fibrils alone (23.03% ± 1.82%, 43.50% ± 2.20%, 23.57% ± 1.17%, and 47.97% ± 3.02%) and in the control group (20.63% ± 4.92%, 41.87% ± 5.05%, 23.27% ± 1.43%, and 41.57% ± 3.64%). Lipopolysaccharide (LPS)‐stimulated BMDCs were selected as the positive control group, in which proportions of CD40, CD80, CD86, and MHC‐II expression in CD11c^+^ cells were 50.43% ± 2.66%, 50.03±3.91%, 48.03±2.37%, and 54.27±5.36%, respectively (Figure 2 and Figure S8). Furthermore, the level of cytokine TNF‐α released by BMDCs stimulated with the SiO_2_@Aβ42 fibril nanovaccines was five‐fold higher than that induced by Aβ42 fibrils alone (Figure S9). Given that TNF‐α is a key indicator of DC activation and migration [33], the nanovaccines‐induced higher level of TNF‐α was consistent with the upregulated expression of surface markers in BMDCs. Collectively, in vitro experimental results demonstrated that SiO_2_@Aβ42 fibril nanovaccines could stimulate the maturation and activation of BMDCs, which were necessary for the induction of a downstream immune response.

**FIGURE 2:**
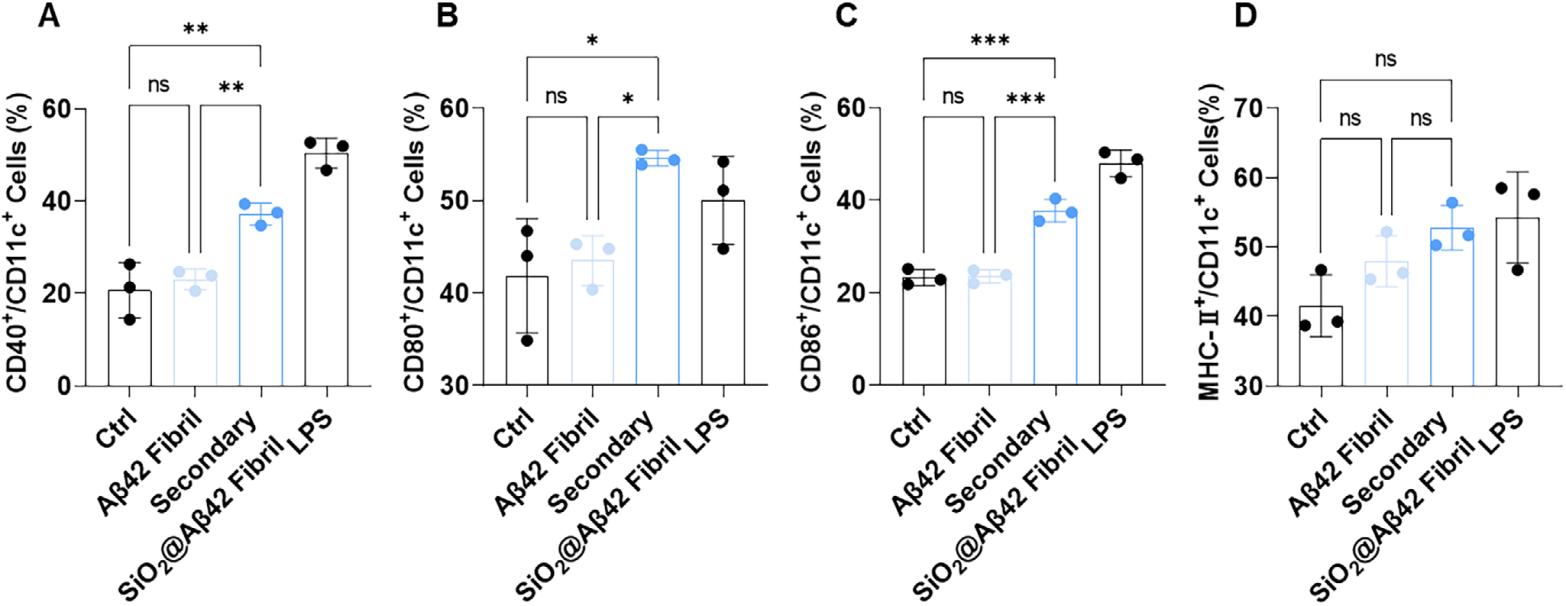
BMDC maturation and activation stimulated with the SiO_2_@Aβ42 fibril nanovaccines. (A–D) Expression of surface markers CD40, CD80, CD86, and MHC‐II in CD11c^+^ cells induced by SiO_2_@Aβ42 fibril nanovaccines at a concentration of 100 µg/mL. BMDCs were stimulated with the nanovaccines for 24 h. The expression of these surface markers was determined using flow cytometry. The monoclonal antibodies (mAbs) used in the experiment included CD16/CD32, Anti‐CD11c Brilliant Violet 605, Anti‐CD40 Super Bright 436, Anti‐CD80 FITC, Anti‐CD86 APCs, and Anti‐I‐A/I‐E PE. Cell culture medium‐treated and Aβ42 fibril‐treated BMDCs served as control groups, *n* = 3.

### 2.3 SiO_2_@Aβ42 Fibril Nanovaccines Ameliorate Behavioral Impairment in APP/PS1 Mice

The immunostimulatory capacity and therapeutic efficacy of the SiO_2_@Aβ42 fibril nanovaccines were determined (Figure 3A,D). WT C57BL/6 mice were used to assess the ability of SiO_2_@Aβ42 fibril nanovaccines to stimulate antibody production, which is necessary for the clearance of Aβ plaques [34, 35]. APP/PS1 transgenic mice exhibiting brain amyloid plaques, motor deficits, and cognitive impairments were used to determine whether SiO_2_@Aβ42 fibril nanovaccines could restore cognitive function in APP/PS1 mice and clear amyloid plaques in the mice's brains [36, 37].

**FIGURE 3:**
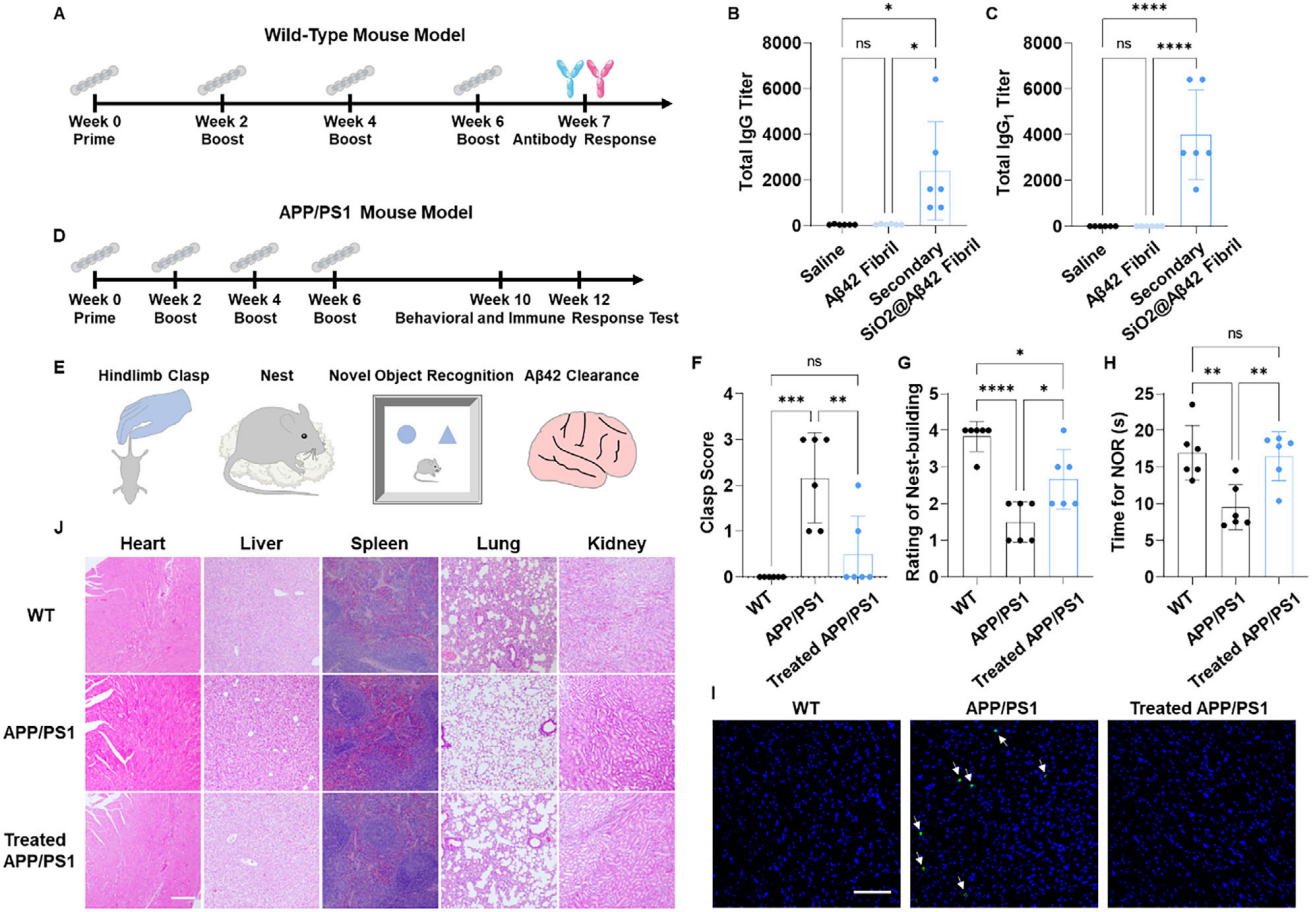
Therapeutic effects of SiO_2_@Aβ42 fibril Nanovaccines in an Alzheimer's Disease Model. (A) Immunization schedule of the nanovaccines in WT mice. Female C57BL/6 mice (6‐week, *n* = 6) were administered the nanovaccines containing 22.5 µg of antigen via subcutaneous (s.c.) injection at Weeks 0, 2, 4, and 6. Serum samples were collected at Week 7. Saline‐treated and antigen‐treated mice served as control groups. (B,C) IgG and IgG_1_, antibody titers in mice's serum were determined by an enzyme‐linked immunosorbent assay (ELISA) *n* = 6. (D) Immunization procedure for the nanovaccines in transgenic mice. Female APP/PS1 mice (7‐month, *n* = 6) were administered with the nanovaccines containing 22.5 µg of antigen via subcutaneous (s.c.) injection at Weeks 0, 2, 4, and 6. Untreated APP/PS1 mice and WT mice served as control groups. Behavioral assessments and immune responses were conducted during Weeks 10–12. (E) Behavioral assessments and immune responses in mice. (F) Score of mice in the hindlimb clasping test. In the hindlimb clasping test, the tails of the mice were grasped and their bodies were suspended in the air for 10 s to assess hindlimb retraction according to the following criteria: both hindlimbs remain fully extended throughout the observation period (0 point); one hindlimb retracts toward the abdomen for over 50% of the observation period (1 point); both hindlimbs alternately and intermittently retract toward the abdomen for over 50% of the observation period (2 points); both hindlimbs fully retract toward the abdomen for over 50% of the observation period (3 points) *n* = 6. (G) Score of mice in the nesting test. In the nesting test, wood shavings bedding (1.5 cm, 65 g) was added to a newly sterilized mouse cage, and two pieces of cotton were placed side by side at the midpoint of the long side of the cage. Mice were individually placed in the cage, and nesting scores were evaluated after 24 h according to the following criteria: cotton remains intact with no obvious chewing marks (1 point); cotton are torn into large fragments scattered throughout the cage or piled beneath the mouse to form a shallow nest (2 points); cotton are torn into small fragments and mixed with wood shavings to form a nest with a noticeable gap, or the nest height is below the mouse's head when the mouse is inside (3 points); cotton are completely torn and mixed with wood shavings to form a nest with no gaps, and the nest height is above the mouse's head when the mouse is inside (4 points) *n* = 6. (H) Duration of exploration in the novel object recognition (NOR) test. In the NOR test, two cylinders were symmetrically placed in a clean box, and mice were allowed to freely explore the box for 2 h. After exploration, mice were allowed to rest for 30 min, during which one cylinder in the box was replaced with a triangular cone. Then the mice were returned to the box, and the time they spent exploring the novel object (triangular cone) was recorded over a 2‐min period *n* = 6. (I) IHC analysis of the Aβ42 levels in the brains of the mice. The brains of mice were processed into 20‐µm slices using a cryostat, and Aβ42 and cell nuclei were stained with Alexa Fluor 488 (green) and DAPI (blue), respectively. The white arrow indicated Aβ42 plaques in the brain. The scale bar is 100 µm. (J) Safety of the SiO_2_@Aβ42 fibril nanovaccines determined by pathological analysis of the major organs. Hearts, livers, spleens, lungs, and kidneys of the mice (female, 7‐month) were collected at week 12 after the behavioral testing, and H&E staining analysis was used to demonstrate the biocompatibility of the SiO_2_@Aβ42 fibril nanovaccines. The scale bar is 200 µm.

First, the immunization regimen for SiO_2_@Aβ42 nanovaccines was determined based on the antibody responses in the serum of WT mice. The results indicated that 4 immunizations induced more significant antibody responses (IgG and IgG_1_) in WT mice compared to 3 immunizations (Figure 3B,C and Figure S10). Thus, the immunization regimen of 4 immunizations was selected. Administration of SiO_2_@Aβ42 fibril nanovaccines to wild‐type (WT) mice elicited higher Aβ42‐specific IgG, and IgG_1_ antibody responses compared to those of Aβ42 fibrils alone or in the control group (Figure 3B,C). Specifically, the serum IgG titers in WT mice treated with SiO_2_@Aβ42 fibril nanovaccines were 2400 ± 1960, significantly higher than those in Aβ42 fibril‐treated mice (53 ± 19) and saline‐treated mice (47 ± 15). Serum IgG_1_ titers in WT mice treated with SiO_2_@Aβ42 fibril nanovaccines were 4000 ± 1789, significantly higher than the 5 ± 0 observed in Aβ42 fibril‐treated and saline‐treated mice. The serum data indicated that SiO_2_@Aβ42 fibril nanovaccines could induce the production of Aβ42‐specific antibodies (IgG and IgG_1_) to ameliorate behavioral impairment. To further determine whether the SiO_2_@Aβ42 fibril nanovaccines could delay the progression of neurodegenerative disease by clearing Aβ plaques through the production of specific antibodies, APP/PS1 transgenic mice were selected as the animal models for therapeutic in vivo experiments. Administration of SiO_2_@Aβ42 fibril nanovaccines to APP/PS1 transgenic mice significantly improved behavioral impairment (Figure 3E). In the hindlimb clasping test, the hindlimb contraction scores in WT mice and untreated APP/PS1 mice were 0.00 ± 0.00 and 2.17 ± 0.90, respectively (Figure 3F and Figure S11). This difference indicated that WT mice exhibited more extended hindlimbs, whereas APP/PS1 mice showed muscle atrophy that impaired their motor function. After treatment with the nanovaccine, the hindlimb contraction score in APP/PS1 mice decreased to 0.50 ± 0.76 (Figure 3F and Figure S11). This suggested that treatment with the SiO_2_@Aβ42 fibril nanovaccines improved motor deficits, alleviated muscle atrophy, and restored motor function in APP/PS1 mice. In the nesting test, the nesting scores of WT mice and untreated APP/PS1 mice were 3.83 ± 0.37 and 1.50 ± 0.50, respectively (Figure 3G and Figure S12). This difference indicated that WT mice had basic cognitive function and exhibited spontaneous nest‐building behavior, whereas APP/PS1 mice showed impaired cognitive function and defective spontaneous nest‐building behavior. After treatment with the nanovaccine, the nesting score of APP/PS1 mice increased to 2.67 ± 0.75 (Figure 3G and Figure S12), indicating a restored nesting ability. These results demonstrated that treatment with the nanovaccines restored the spontaneous nesting behavior and cognitive function of APP/PS1 mice. In the novel object recognition (NOR) test, WT mice and APP/PS1 mice explored novel objects for 16.95 ± 3.40 s and 9.50 ± 2.80 s, respectively (Figure 3H). These results indicated that WT mice had short‐term memory, whereas APP/PS1 mice showed impaired short‐term memory. After treatment with the nanovaccines, the exploration duration of novel objects in APP/PS1 mice was increased to 16.46 ± 3.06 s (Figure 3H), indicating that short‐term memory was restored. These results demonstrated that treatment with the nanovaccines increased the exploration duration of novel objects within a 2‐min experimental period in APP/PS1 mice, indicating a restoration of short‐term memory capacity. The clearance of Aβ plaques in the mice's brains was determined using immunohistochemistry (IHC). The IHC analysis demonstrated that no obvious Aβ42 plaques were detected in the brains of WT mice, whereas obvious Aβ42 plaques were observed in the brains of APP/PS1 mice (Figure 3I). After treatment with the SiO_2_@Aβ42 fibril nanovaccines, the Aβ42 plaques were significantly decreased compared with the untreated mice (Figure 3I). The results from behavioral testing and the detection of brain Aβ42 level synergistically demonstrated that the SiO_2_@Aβ42 fibril nanovaccines restored cognitive function in APP/PS1 mice by clearing Aβ42 plaques. For the safety of the SiO_2_@Aβ42 fibril nanovaccines, the inflammatory infiltration of T cells into the brain was a critical indicator for assessing the safety of Aβ‐based vaccines [38, 39]. Flow cytometry analysis demonstrated that the administration of the nanovaccines did not induce the infiltration of inflammatory T cells into the brain (Figures S13 and S14). Regarding the safety assessment of the SiO_2_@Aβ42 fibril nanovaccines in major organs, H&E staining analysis demonstrated that the nanovaccines did not cause toxicity in key organs, including hearts, livers, spleens, lungs, and kidneys (Figure 3J). These results, along with the absence of T‐cell inflammatory infiltration in the brain and critical organ toxicity, indicated a reliable safety profile for the SiO_2_@Aβ42 fibril nanovaccines. Collectively, in vivo experimental results demonstrated that SiO_2_@Aβ42 fibril nanovaccines could serve as a safe vaccine to improve the behavioral impairment in APP/PS1 mice by clearing Aβ plaques through the production of specific antibodies.

### 2.4 SiO_2_@E7 Fibril Nanovaccines Enhance Anti‐Tumor Effects in Tumor‐Bearing Mice

To examine whether other non‐amyloid antigenic peptides could self‐assemble into nanofibrils to assess the universality of the SiO_2_@fibril nanovaccine platform, SiO_2_ NPs‐coated E7 fibrils (SiO_2_@E7 fibril nanovaccines) were constructed using the human papillomavirus (HPV)‐derived E7 peptide as a representative model antigen. It is important to note that the self‐assembly of E7 peptides alone into nanofibrils has not been reported, and the underlying formation mechanisms remain unclear. Therefore, the formation mechanisms of E7 fibrils were elucidated by investigating the self‐assembly kinetics of E7 peptides at different concentrations (Figure S15). ThT fluorescence analysis confirmed that β‐sheet‐rich aggregates were spontaneously formed after continuous incubation of E7 peptides, a behavior similar to that observed in the self‐assembly of Aβ42. This behavior was due to the ionic environment provided by the HEPES buffer and the inherent hydrophobicity of the E7 peptide (Grand Average of Hydropathy>0) [40, 41, 42], allowing the E7 peptide to undergo a self‐assembly process involving nucleation and elongation. However, at lower concentrations of E7 peptide (such as 0–400 µg/mL), the β‐sheet structure of E7 self‐assembled aggregates collapsed as incubation proceeded, unlike that of Aβ42. This was because of the relatively short sequence of the E7 peptide (9 amino acids), which did not provide a sufficiently dense hydrogen bond network to maintain the β‐sheet structure within the E7 fibrils, leading to its collapse as incubation continued [43, 44]. Conversely, a higher concentration (such as 800 µg/mL) of E7 peptide promoted the formation of a dense network of hydrogen bonds, resulting in more stable E7 fibrils. E7 peptide was first self‐assembled into nanofibrils rich in β‐sheet secondary structure in HEPES buffer solution (Figure 4A and Figure S15), and then APTES and TEOS were sequentially added to prepare the SiO_2_@E7 fibril nanovaccines (Figure 4A and Figure S16). TEM‐based size measurements indicated a uniform width distribution for E7 fibrils and SiO_2_@E7 fibril nanovaccines, with widths of 11 ± 2 and 19 ± 4 nm, respectively (Figure 4B and Table S2). The width of E7 fibrils increased by 1.72 times after the nucleation and growth of SiO_2_ NPs along their surface, similar to that observed for Aβ42 fibrils and SiO_2_@Aβ42 fibril nanovaccines. DLS analysis showed that the hydrodynamic sizes of the E7 fibrils and SiO_2_@E7 fibril nanovaccines were 1274 ± 69 and 595 ± 24 nm, respectively, and their zeta potentials were 5 ± 0 and 31 ± 0 mV, respectively (Table S2). The larger hydrodynamic size of E7 fibrils compared with that of SiO_2_@E7 fibril nanovaccines may be attributed to the aggregation of E7 fibrils caused by their near‐neutral electrical property in deionized H_2_O. EDS analysis confirmed homogeneous co‐localization of Si and O elements within the SiO_2_@E7 fibril nanovaccines (Figure 4C), and ICP‐OES analysis revealed a mass ratio of Si to E7 of 0.59 ± 0.05 in the SiO_2_@E7 fibril nanovaccines (Figure 4D). The results from EDS and ICP‐OES analyses indicated the formation of the SiO_2_ NPs along E7 fibrils.

**FIGURE 4:**
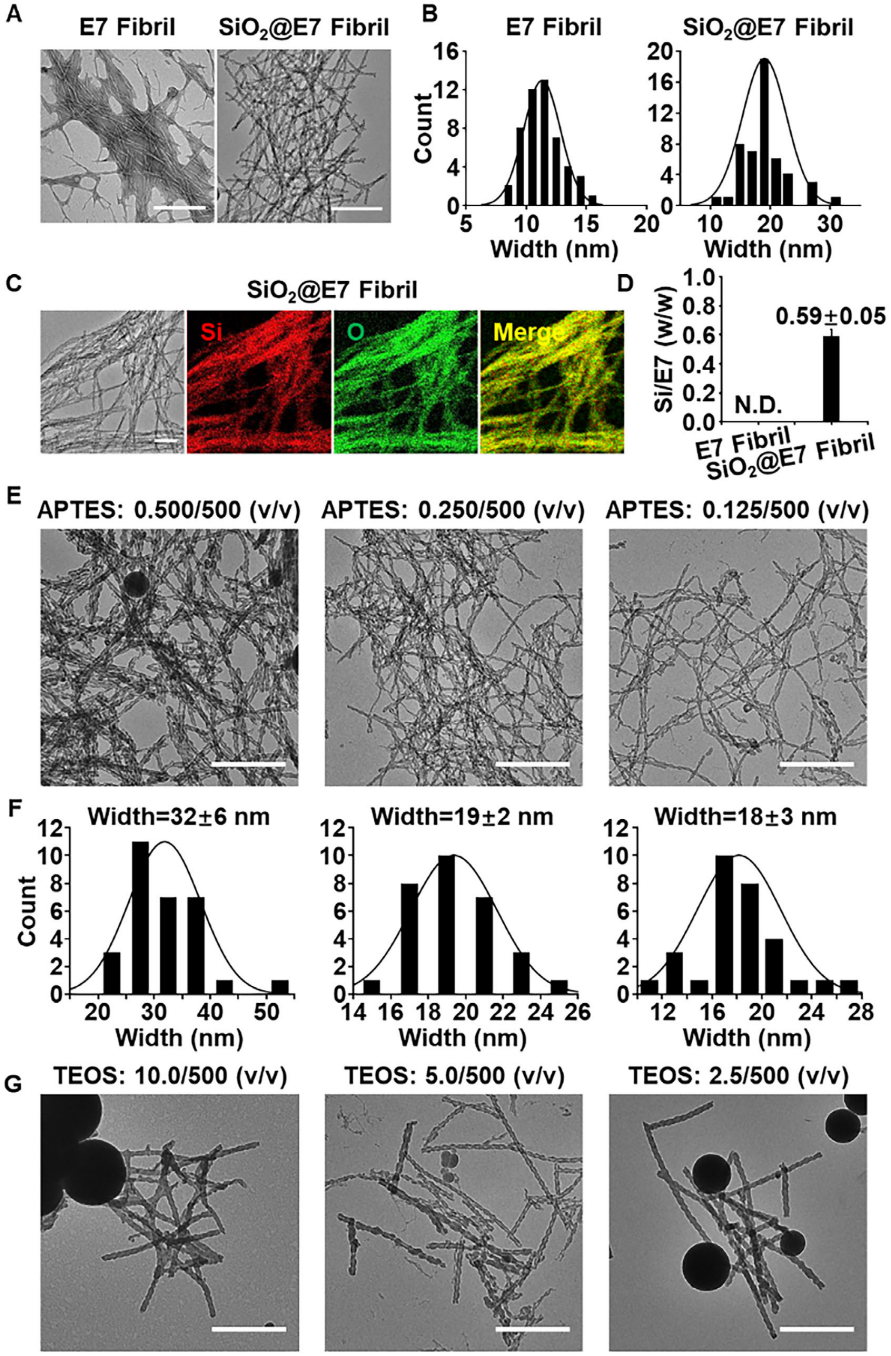
Preparation and characterization of the SiO_2_@Aβ42 fibril nanovaccines. (A) TEM images of the E7 fibrils and SiO_2_@E7 fibril nanovaccines. The scale bar is 500 nm. (B) Width distributions of the E7 fibrils and SiO_2_@E7 fibril nanovaccines. (C) EDS analysis of the co‐localization of Si and O in the SiO_2_@E7 fibril nanovaccines. The scale bar is 200 nm. (D) ICP‐OES analysis of the mass ratios of Si to E7 in the E7 fibrils and SiO_2_@E7 fibril nanovaccines, *n* = 3. (E) TEM images of SiO_2_@E7 fibril nanovaccines prepared under different concentrations of APTES. The scale bar is 500 nm. (F) Average widths of SiO_2_@E7 fibril nanovaccines prepared under different APTES concentrations. (G) TEM images of SiO_2_@E7 fibril nanovaccines prepared under different concentrations of TEOS. The scale bar is 500 nm.

To explore the detailed growth mechanism of the SiO_2_@E7 fibril nanovaccines, the roles of APTES and TEOS in the formation of the SiO_2_@E7 fibril nanovaccines were investigated. APTES served as a nucleating agent for SiO_2_ NPs and determined the width of the SiO_2_@E7 fibril nanovaccines. Lower APTES concentrations promoted the formation of narrower nanovaccines (Figure 4E). In detail, when the concentrations of APTES were 0.500/500 (v/v), 0.250/500 (v/v), and 0.125/500 (v/v), the widths of the SiO_2_@E7 fibril nanovaccines were 32 ± 6, 19 ± 2, and 18 ± 3 nm, respectively (Figure 4F). TEOS served as a growth agent for SiO_2_ NPs and determined whether SiO_2_ NPs could grow completely along the APTES nuclei on the E7 fibrils. Higher TEOS concentrations promoted the generation of free spherical SiO_2_ NPs (Figure 4G). The roles of APTES and TEOS in the formation of the SiO_2_@E7 fibril nanovaccines were consistent with those in forming the SiO_2_@Aβ42 fibril nanovaccines. Additionally, secondary nucleation‐growth of SiO_2_ NPs resulted in wider SiO_2_@E7 fibril nanovaccines (Figure S17A and Table S2). The mass ratio of Si to E7 increased to 7.47 ± 0.18 (Figure S17B), and the width increased to 92 ± 14 nm (Table S2). The hydrodynamic size and zeta potential were 3429 ± 758 nm and 21 ± 1 mV, respectively (Table S2).

The secondary SiO_2_@E7 fibril nanovaccines exhibited negligible cytotoxicity toward BMDCs (Figure S18) at concentrations below 100 ug/mL. The SiO_2_@E7 fibril nanovaccines induced the expression of surface markers on BMDCs. Flow cytometric analysis revealed that the percentages of CD40^+^, CD80^+^, CD86^+^, and MHC II^+^ cells in the CD11c^+^ cell population stimulated with the SiO_2_@E7 fibril nanovaccines were 8.96% ± 0.63%, 75.73% ± 1.96%, 18.43% ± 0.21%, and 52.00% ± 1.90%, respectively. These values were higher than those stimulated with E7 fibrils alone (3.69% ± 0.49%, 57.80% ± 1.36%, 13.23% ± 0.34%, and 40.33% ± 0.63%) and the control group (3.96% ± 0.04%, 59.90% ± 1.12%, 10.14% ± 0.79%, and 37.88% ± 0.40%) (Figure 5A–D and Figure S19). LPS‐stimulated BMDCs were selected as the positive control group, in which proportions of CD40, CD80, CD86, and MHC‐II expression in CD11c^+^ cells were 51.73% ± 5.27%, 87.70% ± 0.83%, 54.90% ± 2.01%, and 64.57% ± 2.22%, respectively (Figure 5A–D and Figure S19). In addition, the SiO_2_@E7 fibril nanovaccines promoted the release of cytokines from BMDCs, including TNF‐α (associated with DCs maturation and migration [33]), IL‐6 (a Th2‐type immune response cytokine [45]), and IL‐12 (a Th1‐type immune response cytokine [46]) (Figure S20A–C). In detail, the concentrations of cytokines TNF‐α, IL‐6, and IL‐12 released by BMDCs stimulated with the SiO_2_@E7 fibril nanovaccines were 283.65 ± 16.57, 427.50 ± 26.70, and 27.00 ± 2.18 pg/mL, respectively. All were significantly higher than those stimulated with E7 fibrils alone (239.38 ± 7.53, 246.90 ± 21.25, and 17.56 ± 1.73 pg/mL, respectively) and the control group (219.79 ± 6.90, 141.51 ± 13.21, and 20.44 ± 0.87 pg/mL, respectively) (Figure S20A–C).

**FIGURE 5:**
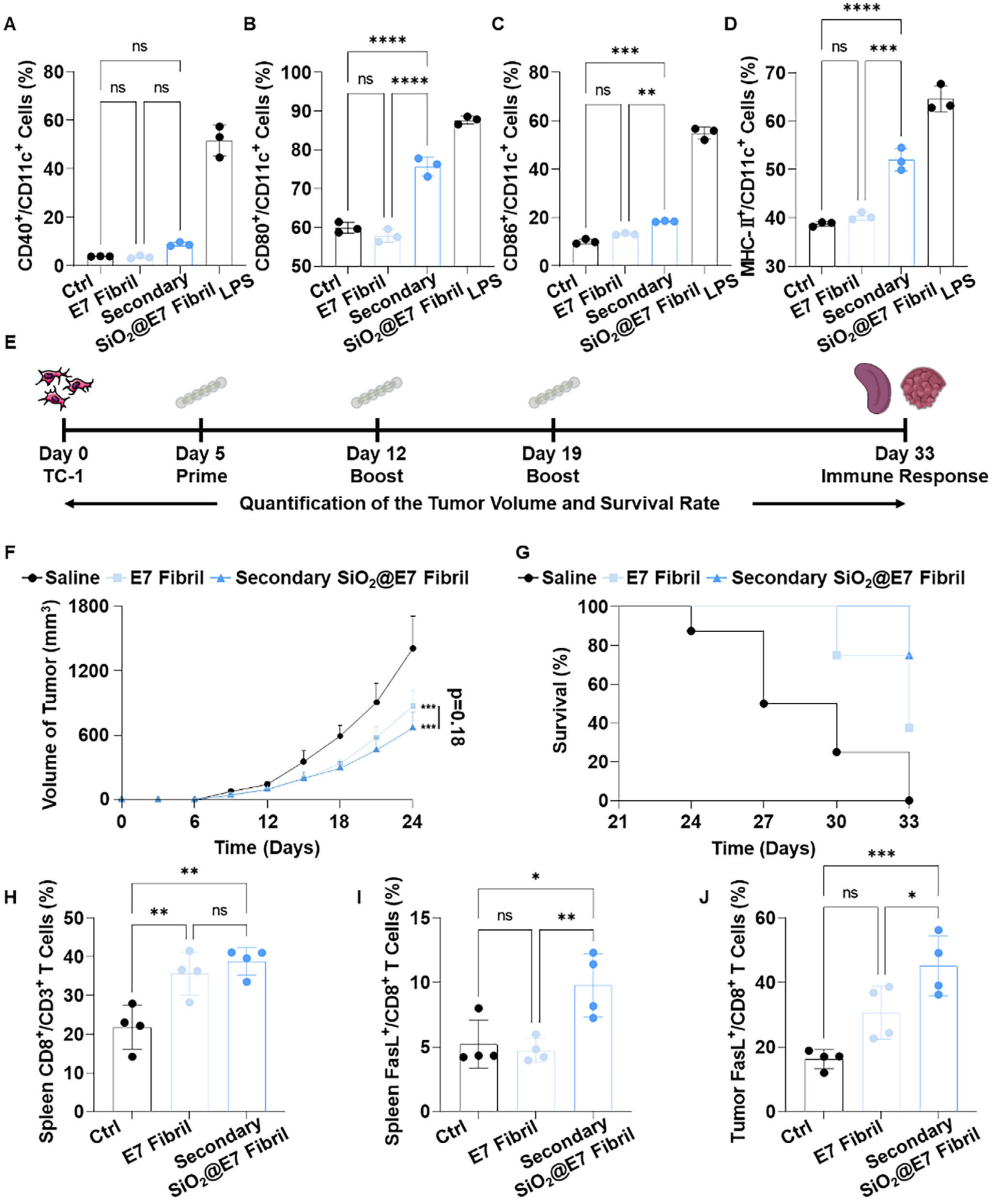
Therapeutic effects of SiO_2_@E7 fibril Nanovaccines in a TC‐1 tumor‐bearing mice model. (A–D) Expression of surface markers CD40, CD80, CD86, and MHC‐II in CD11c^+^ cells induced by the SiO_2_@E7 fibril nanovaccines. BMDCs in these experiments were stimulated with the nanovaccines for 24 h at a concentration of 100 µg/mL. The cells were collected, and the expression of these markers was determined using flow cytometry. Cell culture medium‐treated and E7 fibril‐treated BMDCs served as control groups, *n* = 3. (E) Immunization procedure for the nanovaccines. Female C57BL/6 mice (6‐week, *n* = 8) were inoculated with 2 × 10^5^ TC‐1 cells in the right flank on day 0. Mice were administered with the SiO_2_@E7 fibril nanovaccines containing 10 µg of antigen via intramuscular (i.m.) injection on days 5, 12, and 19. Saline‐treated and antigen‐treated mice served as control groups. Immune responses were evaluated on day 33. (F) Tumor volume (0.5×shortest diameter^2^×longest diameter) growth in mice over 24 days, *n* = 8. (G) Survival rates in mice (mice were sacrificed when the tumor volume >2000 mm^3^) over 33 days, *n* = 8. (H–J) Expression of markers in splenocytes and tumor cells obtained from immunized mice. (H) Percentages of CD8^+^ cells among CD3^+^ T cells in splenocytes. (I) Percentages of FasL^+^ cells among CD8^+^ T cells in splenocytes. (J) Percentages of FasL^+^ cells among CD8^+^ T cells in tumor cells. The expression of the markers was determined using flow cytometry. The mAbs used in the experiment included CD16/CD32, Anti‐CD3 APC/Cy7, Anti‐CD8 APC, and Anti‐FasL PE, *n* = 4.

The anti‐tumor effects of the SiO_2_@E7 fibril nanovaccines were determined in a TC‐1 tumor‐bearing model (Figure 5E). The immunization regimen for SiO_2_@E7 fibril nanovaccines was determined through the immunotherapeutic effects, and the results indicated that 3 immunizations produced better immunotherapeutic effects (tumor volume and survival) in TC‐1 tumor‐bearing mice compared to 2 immunizations (Figure 5F,G and Figure S21). Thus, the immunization regimen of 3 immunizations was selected. The results showed that both SiO_2_@E7 fibril nanovaccines and E7 fibrils significantly slowed tumor growth compared with the control group (Figure 5F and Figures S22 and S23), and the SiO_2_@E7 fibril nanovaccines improved the mouse survival rate (6/8) compared with E7 fibrils (3/8) and the control group (0/8) (Figure 5G). Further mechanistic analysis showed that both the SiO_2_@E7 fibril nanovaccines and E7 fibrils induced the activation of CD8^+^ T cells in spleens, with percentages of 38.80% ± 3.12% and 35.65% ± 4.77%, respectively, higher than that in the control group (21.83% ± 4.91%) (Figure 5H and Figure S24). The SiO_2_@E7 fibril nanovaccines also induced higher levels of FasL^+^ CTL in spleens (9.78% ± 2.12%), higher than those induced by E7 fibrils alone and the control group (4.75% ± 0.78% and 5.21% ± 1.61%, respectively) (Figure 5I and Figure S24). In addition, the nanovaccines promoted infiltration of FasL^+^ CTL into tumors (45.13% ± 7.99%), higher than those induced by free E7 fibrils and the control group (30.63% ± 7.13% and 16.25% ± 2.57%, respectively) (Figure 5J). Collectively, it was demonstrated that SiO_2_@fibril nanovaccines could serve as a universal immunotherapy strategy targeting multiple diseases (including AD and HPV) based on corresponding immunogenic antigen peptides (Aβ42 and E7).

## 3 Discussion

In this study, we developed a safe and universal nanovaccine platform composed of uniformly silica nanoparticle (SiO_2_ NP)‐coated antigenic nanofibrils (SiO_2_@fibril nanovaccines). Antigenic peptides self‐assembled into nanofibrils without the incorporation of foreign β‐sheet‐forming sequences, followed by SiO_2_ NP formation along fibril surfaces via a nucleation‐growth process. Using Aβ42 and as model antigens, SiO_2_@fibril nanovaccines exhibited good biocompatibility and efficiently promoted the maturation and activation of BMDCs, as evidenced by upregulated surface markers (CD40, CD80, CD86, and MHC‐II) and enhanced cytokine secretion. In vivo, SiO_2_@Aβ42 nanovaccines elicited robust humoral immune responses, reduced cerebral Aβ42 plaque burden without inducing inflammatory T‐cell infiltration, and restored motor and cognitive function in APP/PS1 mice. Meanwhile, SiO_2_@E7 nanovaccines strong CTL responses, suppressed tumor growth, and survival in TC‐1 tumor‐bearing mice.

It has been shown that the self‐assembly of antigenic peptides commonly depended on covalently conjugating antigenic sequences with β‐sheet‐forming sequences, and the conjugated products self‐assembled into nanofibrils to act as a self‐delivery system [17, 18, 19, 20]. The major difference between our design and previously published studies was that the self‐assembly conditions for the antigenic peptides in our study were rationally designed based on the peptides’ intrinsic properties, such as surface hydrophobicity and isoelectric point. It is well known that the self‐assembly of peptides into nanofibrils follows typical nucleation‐and‐elongation kinetics, so the peptides must overcome intermolecular electrostatic repulsion and utilize their intrinsic hydrophobicity to first assemble into oligomers (nuclei), after which they stack continuously along the nuclei to grow into mature nanofibrils [47, 48, 49]. The wide variation in antigenic peptide sequences led to significant differences in surface hydrophobicity and charge. Low hydrophobicity and high surface charge were unfavorable to self‐assembly, which is why such antigens required well‐established β‐sheet‐forming sequences [50]. For our designed nanovaccine platform, we first quantified antigen peptide hydrophobicity using the grand average of hydropathy (GRAVY), with more positive GRAVY values indicating stronger hydrophobicity. Then, suitable self‐assembly conditions, such as ionic strength and pH, were selected accordingly. Higher ionic strength and a pH close to the isoelectric point facilitated nucleation by weakening electrostatic repulsion [50]. Specifically, the GRAVY values of Aβ42 and E7 were 0.20 and 0.01, respectively, with values above 0 indicating relatively hydrophobic peptides. Their isoelectric points were 5.43 and 9.35, respectively, suggesting that near‐neutral conditions were favorable to their self‐assembly. Therefore, 10 mm HEPES buffer (pH 7.0) was chosen. ThT and TEM results demonstrated the formation of nanofibrils rich in β‐sheet secondary structure after continuous incubation of Aβ42 and E7 peptides (Figures 1B and 4A, Figures S1 and S15). Collectively, by quantifying peptide hydrophobicity and optimizing self‐assembly conditions accordingly, the construction of self‐delivery systems was greatly simplified, and the introduction of exogenous β‐sheet‐forming sequences into antigenic peptides capable of self‐assembling into nanofibrils was avoided.

In addition to addressing the challenges of self‐assembling antigenic peptides capable of forming nanofibrils, this study also addressed the difficulty of selecting delivery systems and vaccine adjuvants for peptide vaccines. This is because delivery systems/vaccine adjuvants usually exert their effects through interactions with antigens, while the wide range of intrinsic properties of antigenic peptides (such as hydrophobicity and surface charge) makes it difficult to select a universal delivery system/vaccine adjuvant applicable to most antigens [51, 52]. In the nanovaccines designed in this study, SiO_2_ NPs were formed by nucleation and grew along the surface of antigenic peptide fibrils. Importantly, this approach was not limited to specific types of antigenic peptide fibrils but rather represented a universal strategy. This is because the nucleation of SiO_2_ NPs in this strategy relied on the interaction between positively charged APTES and peptides, while the neutral environment and the large number of negatively charged carboxyl groups in the peptide chains provided sufficient interaction sites for APTES, promoting the formation of SiO_2_ NP nuclei [26]. Notably, co‐incubation of APTES with antigenic peptides (especially those containing Glu and Asp) could theoretically lead to amide bond formation to affect the antigen immunogenicity, but this reaction requires EDC/NHS‐mediated carboxyl activation under acidic conditions [53, 54], which are not met during nanovaccine preparation. Moreover, enzyme‐linked immunosorbent assay (ELISA) results confirmed that the antigen released after silica dissolution was recognized by antibodies to a similar extent as the unencapsulated antigen (Figure S4). Therefore, co‐incubation of APTES with antigenic peptides and the formation of silica coating did not affect the antigen immunogenicity. Subsequently, TEOS acted as a growth agent to facilitate the continuous formation of SiO_2_ NPs along the APTES nuclei. The roles of APTES and TEOS were verified in this study on the formation mechanisms of SiO_2_@Aβ42 fibril nanovaccines and SiO_2_@E7 fibril nanovaccines (Figures 1F,G and 4E–G). In conclusion, the nucleation and growth of SiO_2_ NPs along the antigenic peptide fibrils were not limited to specific types of antigenic fibrils. Instead, they represented a universal construction process for antigenic peptide delivery systems/vaccine adjuvants of antigenic peptide fibrils.

Following injection, the SiO_2_@fibril nanovaccines are internalized and processed by APCs such as BMDCs. On the one hand, the nanovaccines are delivered to the lysosomes of APCs, where the antigenic peptide fibrils coated with SiO_2_ NPs are released due to acid‐induced silica dissolution [26, 55, 56]. On the other hand, the antigenic peptides are degraded by proteases within the APCs into short peptide fragments that can be recognized by T cell receptors (TCRs) (since intact proteins cannot be directly recognized by TCRs), and the resulting fragments depend on the intrinsic sequence of the full‐length antigenic peptide [57, 58]. These processed short peptide fragments are then presented to T cells (such as CD4^+^ Th2, CD4^+^ Th1, and CD8^+^ T cells) to induce adaptive immune responses [59, 60]. Meanwhile, innate immune responses are also activated after vaccine injection, in addition to antigen internalization and processing. The experimental results demonstrated that the SiO_2_ NP‐coated antigenic peptide fibrils were more effective than uncoated antigenic peptide fibrils at stimulating the maturation and activation of bone marrow‐derived dendritic cells (BMDCs), a representative type of APCs. These maturation and activation included the expression of surface markers (such as CD40, CD80, CD86, and MHC‐II) and the release of cytokines (such as TNF‐α) (Figures 2 and 5A–D, and Figures S9 and S20). The maturation and activation of BMDCs are critical for efficient antigen processing and presentation, thereby inducing robust adaptive immune responses. Additionally, previously published studies have also confirmed that SiO_2_ NP‐adjuvanted vaccines more effectively induce the expression of surface markers on APCs and the release of cytokines compared with unadjuvanted vaccines [61, 62, 63]. Collectively, the results of our experiments and published studies demonstrate that the SiO_2_@fibril nanovaccines platform is flexible, stemming from the universal immune activation mechanism of SiO_2_ NPs as vaccine adjuvants. Overall, the SiO_2_@fibril nanovaccine platform was capable of eliciting balanced cellular and humoral immune responses (Figures 3 and 5). This is because, as silica‐coated vaccines, they not only exhibit an excellent ability to stimulate innate responses (such as the expression of surface markers and the release of cytokines), but also promote the antigen escape from lysosomes, which could favor the cross‐presentation of the coated antigen [26]. Although conventional alum‐based vaccines have also been reported to stimulate the innate responses, they are unable to induce antigen cross‐presentation, which limits them to inducing only humoral immunity mediated by antibody responses [64]. This comparison demonstrates the superiority of SiO_2_@fibril nanovaccine platform in eliciting a more comprehensive immune response.

## 4 Conclusion

In summary, we developed a SiO_2_@fibril nanovaccine platform suitable for antigenic peptides capable of self‐assembling into nanofibrils, nucleating silica nanoparticles directly onto self‐assembled antigenic nanofibrils. By bypassing the need for exogenous β‐sheet‐forming motifs, this modular approach proved effective for both amyloid‐β(1‐42) (Aβ42) and human papillomavirus type 16 E7 (E7) antigens. In vitro, the nanovaccines significantly enhanced BMDC maturation and cytokine secretion. In vivo, SiO_2_@Aβ42 fibril nanovaccines cleared amyloid plaques and reversed cognitive deficits in APP/PS1 mice without inducing neuroinflammation, whereas SiO_2_@E7 fibril nanovaccines triggered robust CTL responses that suppressed tumor growth and extended survival. These results demonstrate that the SiO_2_@fibril architecture is a safe, potent, and universal strategy for enhancing the immunogenicity of diverse peptide antigens.

## 5 Experimental Section

### 5.1 Materials and Reagents

Aβ42 (sequence: DAEFRHDSGYEVHHQKLVFFAEDVGSNKGAIIGLMVGGVVIA, purity>95%) peptide was purchased from GL Biochem (Shanghai, China). E7 (sequence: RAHYNIVTF, purity>98%) peptide was purchased from GenScript (Nanjing, China). (3‐Aminopropyl) triethoxysilane (APTES) was purchased from Macklin (Shanghai, China). Tetraethyl orthosilicate (TEOS) was purchased from Aladdin (Shanghai, China). All chemical reagents were used directly without further purification.

### 5.2 Preparation of SiO_2_@Aβ42/E7 Fibril Nanovaccines

In the synthesis of SiO_2_@Aβ42 fibril nanovaccines, Aβ42 monomers (200 µm) were first incubated in 10 mm HEPES buffer at 37°C for 12 h to completely self‐assemble into Aβ42 fibrils. Next, APTES and TEOS were sequentially added to the Aβ42 fibril solution at concentrations of 0.5/500 (v/v) and 10/500 (v/v) to promote the nucleation and growth of SiO_2_ nanoparticles (NPs) on the surface of Aβ42 fibrils. The final mixture was aged at 25°C. In the synthesis of SiO_2_@E7 fibril nanovaccines, E7 peptide (200 µg/mL) was first incubated for 3 h to form E7 fibrils. Then, APTES and TEOS were sequentially added to the E7 fibril solution at concentrations of 0.25/500 (v/v) and 5/500 (v/v), respectively. The final mixture was also aged at 25°C. All NPs were washed with deionized water at least three times before further experiments.

### 5.3 Characterizations of SiO_2_@Aβ42/E7 Fibril Nanovaccines

The morphologies of SiO_2_@Aβ42/E7 fibril nanovaccines and Aβ42/E7 fibrils were observed by transmission electron microscopy (TEM) (JEM‐2000EX, JEOL, Japan) at an acceleration voltage of 120 kV. In addition, Aβ42 and E7 fibrils were negatively stained using phosphotungstic acid solution (2%) before observation. The widths of SiO_2_@Aβ42/E7 fibril nanovaccines and Aβ42/E7 fibrils were measured using ImageJ based on their TEM images. Qualitative elemental distribution analysis of SiO_2_@Aβ42/E7 fibril nanovaccines was performed by energy dispersive X‐ray spectroscopy (EDS). Quantitative analysis of silicon in SiO_2_@Aβ42/E7 fibril nanovaccines and Aβ42/E7 fibrils was determined by inductively coupled plasma optical emission spectroscopy (ICP‐OES). Hydrodynamic size, polydispersity (PDI), and zeta potential of SiO_2_@Aβ42/E7 fibril nanovaccines and Aβ42/E7 fibrils were measured by dynamic light scattering (DLS, Brookhaven, 90 plus PALS, USA).

### 5.4 Determination of BMDCs Maturation

BMDC maturation was determined by surface marker expression analyzed by flow cytometry (BD FACSymphony A1, USA). The monoclonal antibodies (mAbs) included non‐specific binding blocking mAbs CD16/CD32, and 5 flow cytometry mAbs, such as Anti‐CD11c Brilliant Violet 605, Anti‐CD40 Super Bright 436, Anti‐CD80 FITC, Anti‐CD86 APCs, and Anti‐I‐A/I‐E PE. BMDCs were treated using SiO_2_@Aβ42 fibril nanovaccines or SiO_2_@E7 fibril nanovaccines for 24 h. The cells were then collected and incubated with CD16/CD32 mAbs and five flow cytometry mAbs for 10 and 30 min, respectively. The results were analyzed using FlowJo software.

### 5.5 Animal Vaccinations

Wild‐type female C57BL/6 mice (6–8 weeks) and APP/PS1 transgenic female C57BL/6 mice (7 months) were purchased from Changsheng Biotechnology (Liaoning, China). All animals were housed under standard laboratory conditions in accordance with guidelines approved by the Institutional Animal Care and Use Committee of Dalian University of Technology (DUTSCE220301_08). For the wild‐type mice injected with SiO_2_@Aβ42 fibril nanovaccines, Secondary SiO_2_@Aβ42 fibrils containing 22.5 µg of Aβ42 were subcutaneously injected via an immunization protocol consisting of four doses at 2‐week intervals. Mice were sacrificed 1 week after the final immunization. Aβ42‐fibrils‐injected and saline‐injected mice were used as controls. For the APP/PS1 transgenic mice injected with SiO_2_@Aβ42 fibril nanovaccines, Secondary SiO_2_@Aβ42 fibrils containing 22.5 µg of Aβ42 were subcutaneously injected via an immunization protocol consisting of four doses at 2‐week intervals. Four weeks after the final immunization, the mice were subjected to behavioral and immune response assessment. Unvaccinated APP/PS1 mice and wild‐type mice were used as controls. For the wild‐type mice injected with SiO_2_@E7 fibril nanovaccines, 2 × 10^5^ TC‐1 cells were subcutaneously injected into the right flank of the mice. Five days after tumor cell inoculation, Secondary SiO_2_@E7 fibrils containing 10 µg E7 were intramuscularly administered via an immunization protocol consisting of three doses at 1‐week intervals. Tumor volume (0.5×shortest diameter^2^×longest diameter) and survival rate of the mice were monitored (mice were sacrificed when the tumor volume >2000 mm^3^). All mice were sacrificed 2 weeks after the final immunization. E7 fibrils‐injected and saline‐injected mice were used as controls.

### 5.6 Behavioral Assessments of the Animals

In the hindlimb clasping test, the tails of the mice were grasped and their bodies were suspended in the air for 10 s to assess hindlimb retraction according to the following criteria: both hindlimbs remain fully extended throughout the observation period (0 points); one hindlimb retracts toward the abdomen for over 50% of the observation period (1 point); both hindlimbs alternately and intermittently retract toward the abdomen for over 50% of the observation period (2 points); both hindlimbs fully retract toward the abdomen for over 50% of the observation period (3 points).

In the nesting test, wood shavings bedding (1.5 cm, 65 g) was added to a newly sterilized mouse cage, and two pieces of cotton were placed side by side at the midpoint of the long side of the cage. Mice were individually placed in the cage, and nesting scores were evaluated after 24 h according to the following criteria: cotton remains intact with no obvious chewing marks (1 point); cotton are torn into large fragments scattered throughout the cage or piled beneath the mouse to form a shallow nest (2 points); cotton are torn into small fragments and mixed with wood shavings to form a nest with a noticeable gap, or the nest height is below the mouse's head when the mouse is inside (3 points); cotton are completely torn and mixed with wood shavings to form a nest with no gaps, and the nest height is above the mouse's head when the mouse is inside (4 points).

In the Novel Object Recognition (NOR) test, a clean box (60 cm × 40 cm × 30 cm) was prepared and two cylinders were symmetrically placed in the box, mice were transferred to the box and allowed to freely explore for 2 h. After exploration, mice were returned to their cage and allowed to rest for 30 min, and one cylinder in the box was replaced with a triangular cone during this period. Then the mice were returned to the box, and the time they spent exploring the novel object (triangular cone) was recorded over a 2‐min period.

### 5.7 Statistical Analysis

In all experiments, the experimental data for each group included at least three replicates, and each experiment was repeated at least twice. Data were presented as mean values ± SD. The statistical significance of all data was determined by one‐way ANOVA.

## Author Contributions

**Zhili Wang**: investigation. **Xuecheng Yang**: software, data curation, validation, investigation, project administration, writing – original draft, supervision, conceptualization, methodology, visualization. **Min Li**: conceptualization, methodology, investigation. **Zhiying Yao**: investigation, data curation, software. **Wendi Shi**: investigation. **Bingbing Sun**: conceptualization, funding acquisition, supervision, methodology, project administration, formal analysis, resources, writing – review and editing. **Yu Hu**: investigation.

## Funding

This work was supported by the National Natural Science Foundation of China (U22A20455) and the National Key Research and Development Program of China (2022YFC2304305 and 2025YFC2311603).

## Conflicts of Interest

The authors declare no conflicts of interest.

## Supporting information




**Supporting File**: advs76636‐sup‐0001‐SuppMat.doc.

## Data Availability

The data that support the findings of this study are available from the corresponding author upon reasonable request.
