# Supplementary material for: Self‐Assembly of Antigenic Peptide Nanofibrils Templates the Growth of Silica Nanoparticles for Nanovaccines
Source: Adv Sci (Weinh). 2026 Jul 14:e76636. Online ahead of print. doi: 10.1002/advs.76636 (PMC13366373; doi:10.1002/advs.76636)
Supplement: Supplementary file 1 — Supporting File: advs76636‐sup‐0001‐SuppMat.doc. [file ADVS-9999-e76636-s001.doc]

**Supporting Information**

Self-Assembly of Antigenic Peptide Nanofibrils Templates the Growth of Silica Nanoparticles for Nanovaccines

Xuecheng Yang, Min Li, Zhiying Yao, Yu Hu, Zhili Wang, Wendi Shi and Bingbing Sun*

Xuecheng Yang, Min Li, Zhiying Yao, Yu Hu, Zhili Wang, Wendi Shi and Bingbing Sun

^1^School of Chemical Engineering, Dalian University of Technology, 2 Linggong Road, 116024, Dalian, China

^2^State Key Laboratory of Fine Chemicals, School of Chemical Engineering, Dalian University of Technology, 2 Linggong Road, 116024, Dalian, China

^3^Frontiers Science Center for Smart Materials Oriented Chemical Engineering, Dalian University of Technology, 2 Linggong Road, 116024, Dalian, China
E-mail: bingbingsun@dlut.edu.cn

**
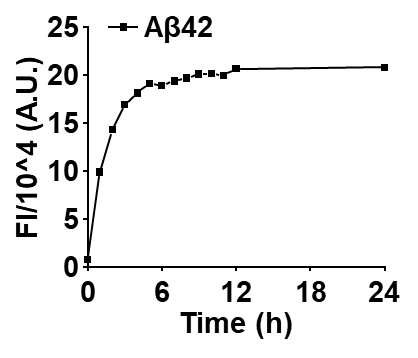
**

**Figure S1. Fibrillation kinetics of the antigen peptide Aβ42.** The fibrillation kinetics of the Aβ42 peptides were determined using a Thioflavin T (ThT) fluorescence analysis. Aβ42 peptide at a concentration of 50 μM was incubated in 10 mM of HEPES buffer at 300 rpm and 37 °C. The fluorescence intensity of Aβ42 was measured at an excitation (E_x_) wavelength of 440 nm and an emission (E_m_) wavelength of 485 nm.

**
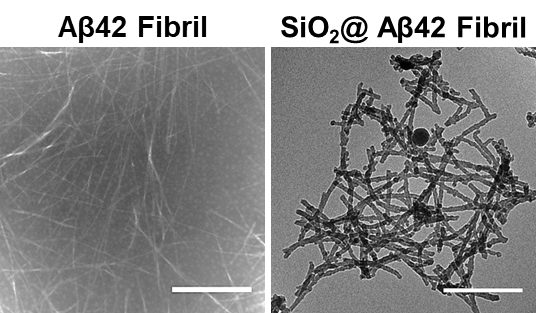
**

**Figure S2.** TEM images of the Aβ42 fibrils and SiO_2_@Aβ42 fibril nanovaccines. The scale bar is 500 nm.

**
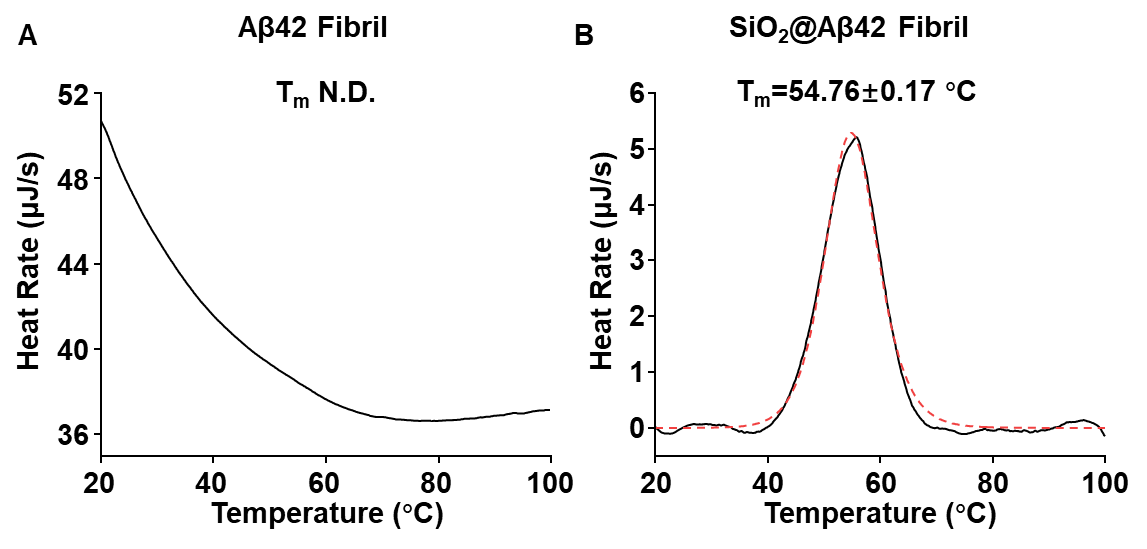
**

**Figure S3. Differential Scanning Calorimetry (DSC) analysis of the Aβ42 fibrils and SiO_2_@Aβ42 fibril nanovaccines.** Aβ42 fibrils and SiO_2_@Aβ42 fibril nanovaccines at a concentration of 200 μg/mL were heated from 20 to 100 °C in the sample cell, respectively. The thermal profiles (solid lines) and peak fitting (dashed lines) during the heating process were recorded, and the profiles of their buffers were subtracted as background.


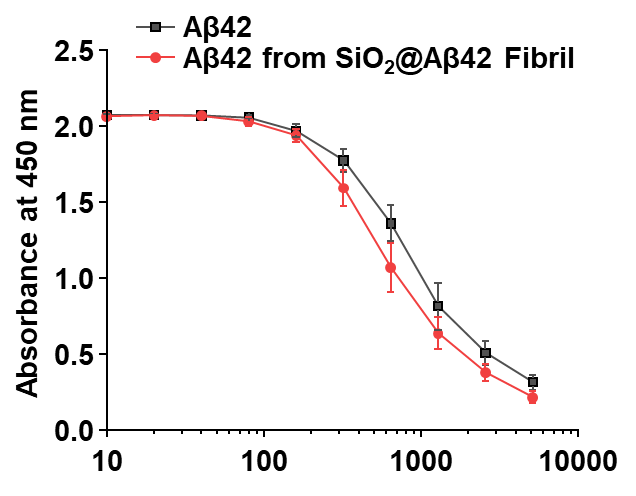


**Figure S4.** **Immunogenicity of released Aβ42 was determined by an enzyme-linked immunosorbent assay (ELISA) analysis.** Polyclonal antibody reactivity to released Aβ42 from SiO_2_@Aβ42 fibril nanovaccines in carbonate buffer.

**
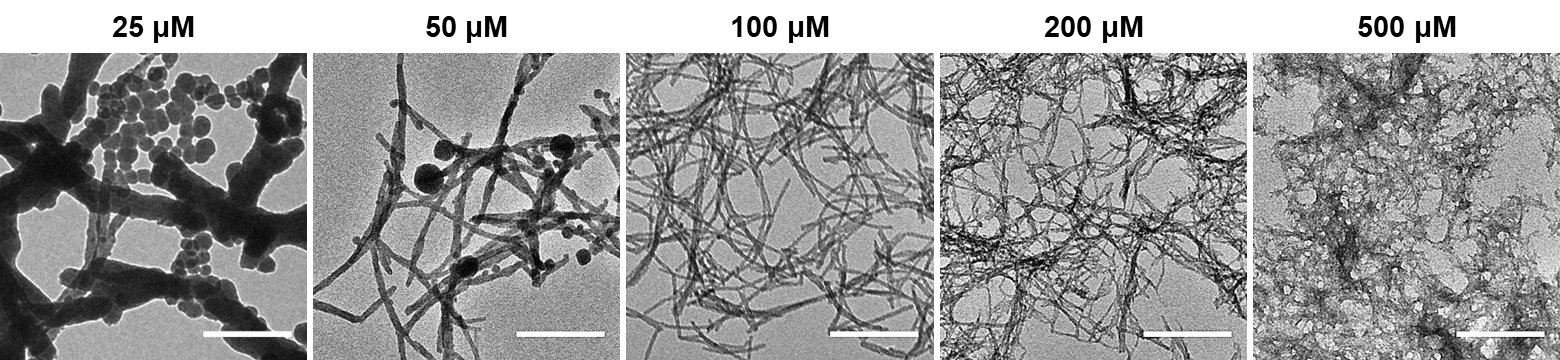
**

**Figure S5.** TEM images showing the effects of Aβ40 concentrations on the morphologies of the SiO_2_@Aβ40 fibril NPs. The scale bar is 500 nm.

**
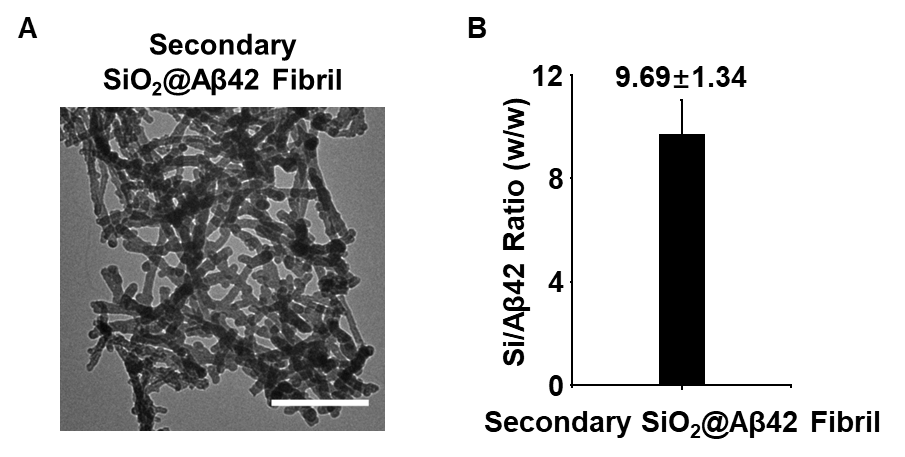
**

**Figure S6. Characterization of the SiO_2_@Aβ42 fibril nanovaccines prepared by the secondary nucleation-growth strategy.** (A) TEM images of the secondary SiO_2_@ Aβ42 fibril nanovaccines. The scale bar is 500 nm. (B) ICP-OES analysis of the mass ratios of Si to Aβ42 in the secondary SiO_2_@ Aβ42 fibril nanovaccines. n=3.

**Figure S7. Biocompatibility of SiO_2_@Aβ42 fibril nanovaccines.** Cell viability of the BMDCs stimulated with the SiO_2_@Aβ42 fibril nanovaccines at concentrations of 50 and 100 μg/mL. The cell viability of BMDCs was measured using an MTS assay kit.

**
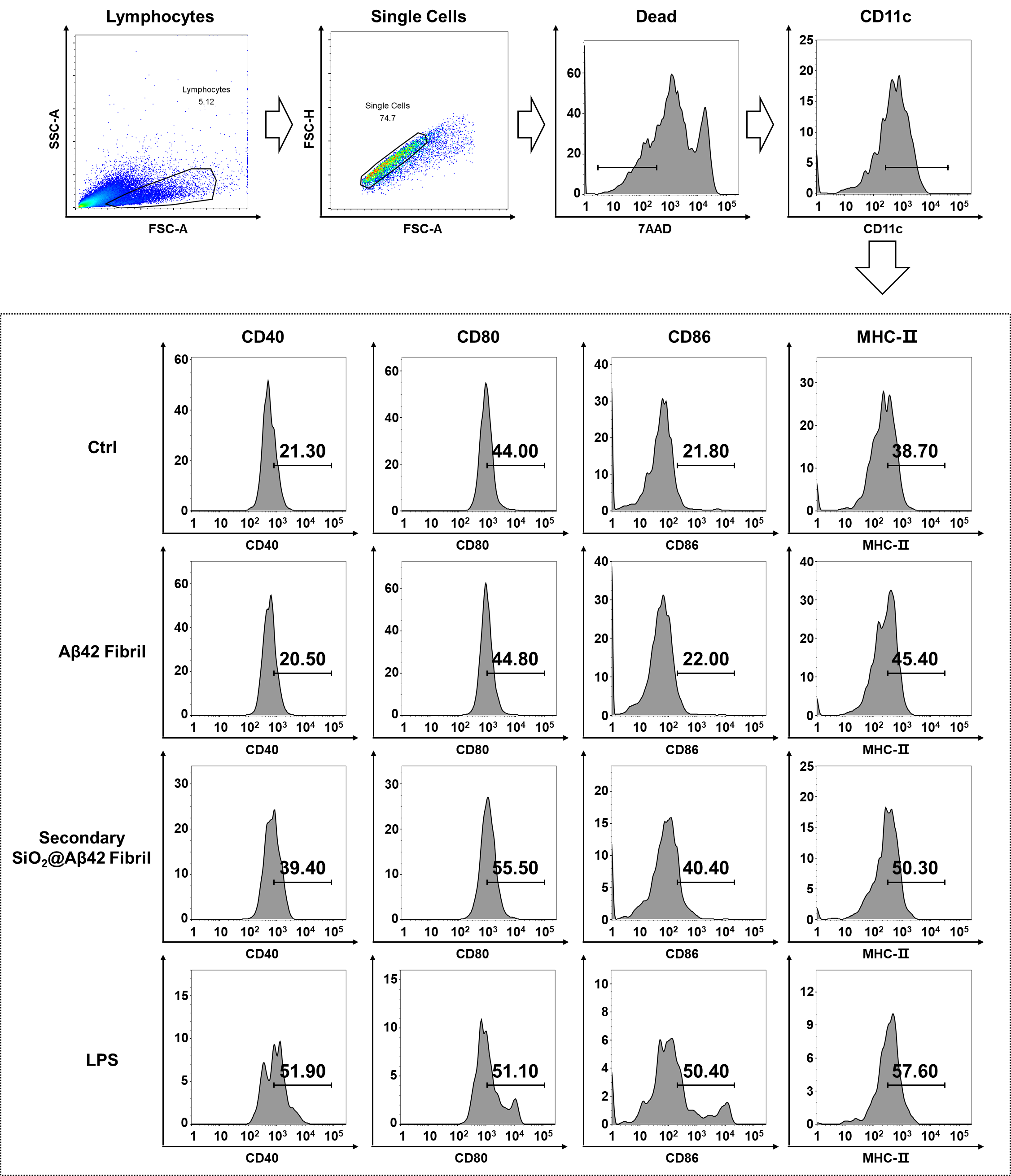
**

**Figure S8. Maturation and activation of BMDCs.** BMDCs were stimulated with the SiO_2_@Aβ42 fibril nanovaccines for 24 hours at a concentration of 100 μg/mL. The cells were then collected, and the expression of the surface markers CD40, CD80, CD86, and MHC-II on BMDCs was measured using flow cytometry. The monoclonal antibodies (mAbs) used in the experiment included CD16/CD32, Anti-CD11c Brilliant Violet™ 605, Anti-CD40 Super Bright™ 436, Anti-CD80 FITC, Anti-CD86 APCs, and Anti-I-A/I-E PE. Cell culture medium-treated and Aβ42 fibril-treated BMDCs served as control groups. n=3. Values shown in the graphs indicated percentages of positive cells in the representative samples.

**Figure S9. The cytokine TNF-α released from BMDCs.** BMDCs were stimulated with the SiO_2_@Aβ42 fibril nanovaccines for 24 hours at a concentration of 100 μg/mL. The culture supernatants were then collected, and TNF-α concentrations were measured using an enzyme-linked immunosorbent assay (ELISA). Cell culture medium-treated and Aβ42 fibril-treated BMDCs served as control groups. n=3.

**
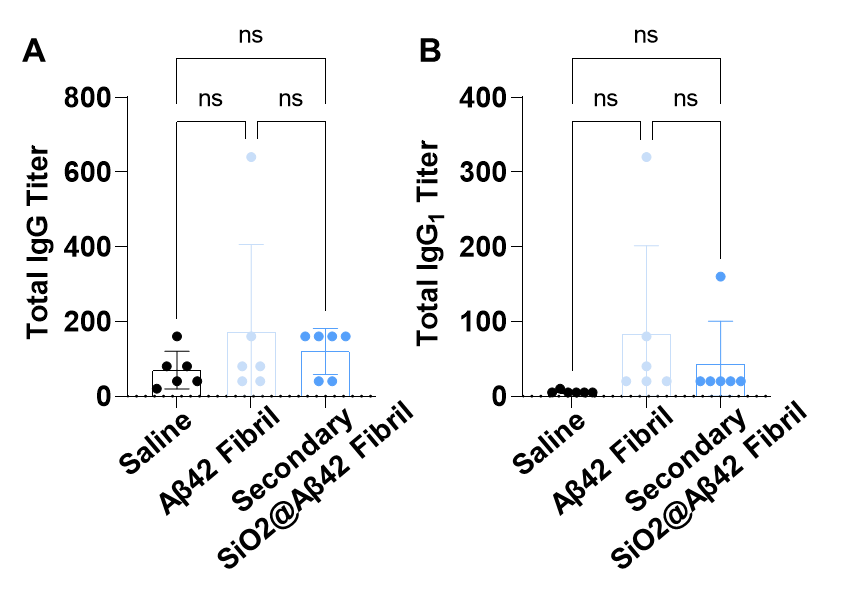
**

**Figure S10.** **Optimization of the vaccination regimen for SiO_2_@Aβ42 fibril nanovaccines.** (A-B) IgG and IgG_1_ antibody titers in mice serum after 3 immunizations with SiO_2_@Aβ42 fibril nanovaccines, determined by enzyme-linked immunosorbent assay (ELISA).

**
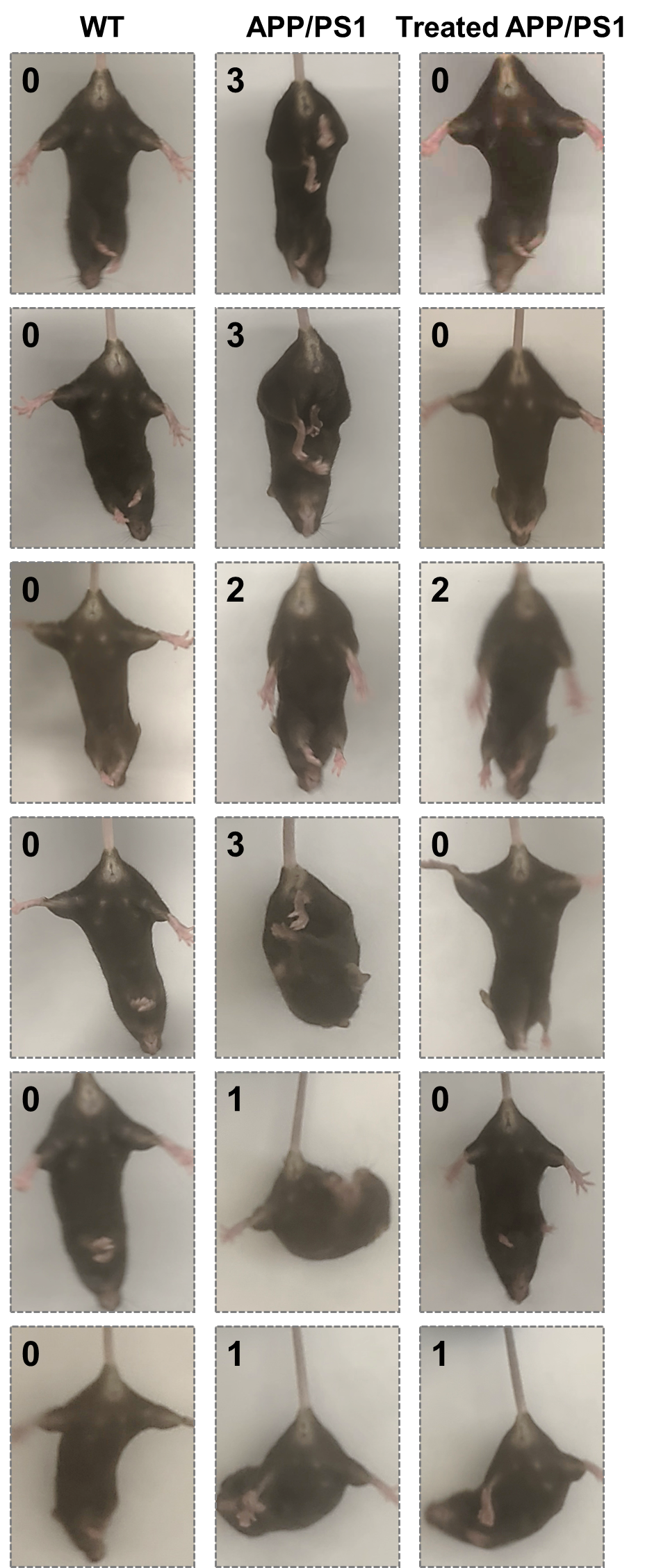
**

**Figure S11. Representative images of mice during the hindlimb clasping test.** APP/PS1 mice (female, 7-month, n=6) were used in the hindlimb clasping test. In the hindlimb clasping test, the tails of the mice were grasped and their bodies were suspended in the air for 10 seconds to assess hindlimb retraction according to the following criteria: both hindlimbs remain fully extended throughout the observation period (0 point); one hindlimb retracts toward the abdomen for over 50% of the observation period (1 point); both hindlimbs alternately and intermittently retract toward the abdomen for over 50% of the observation period (2 points); both hindlimbs fully retract toward the abdomen for over 50% of the observation period (3 points).

**
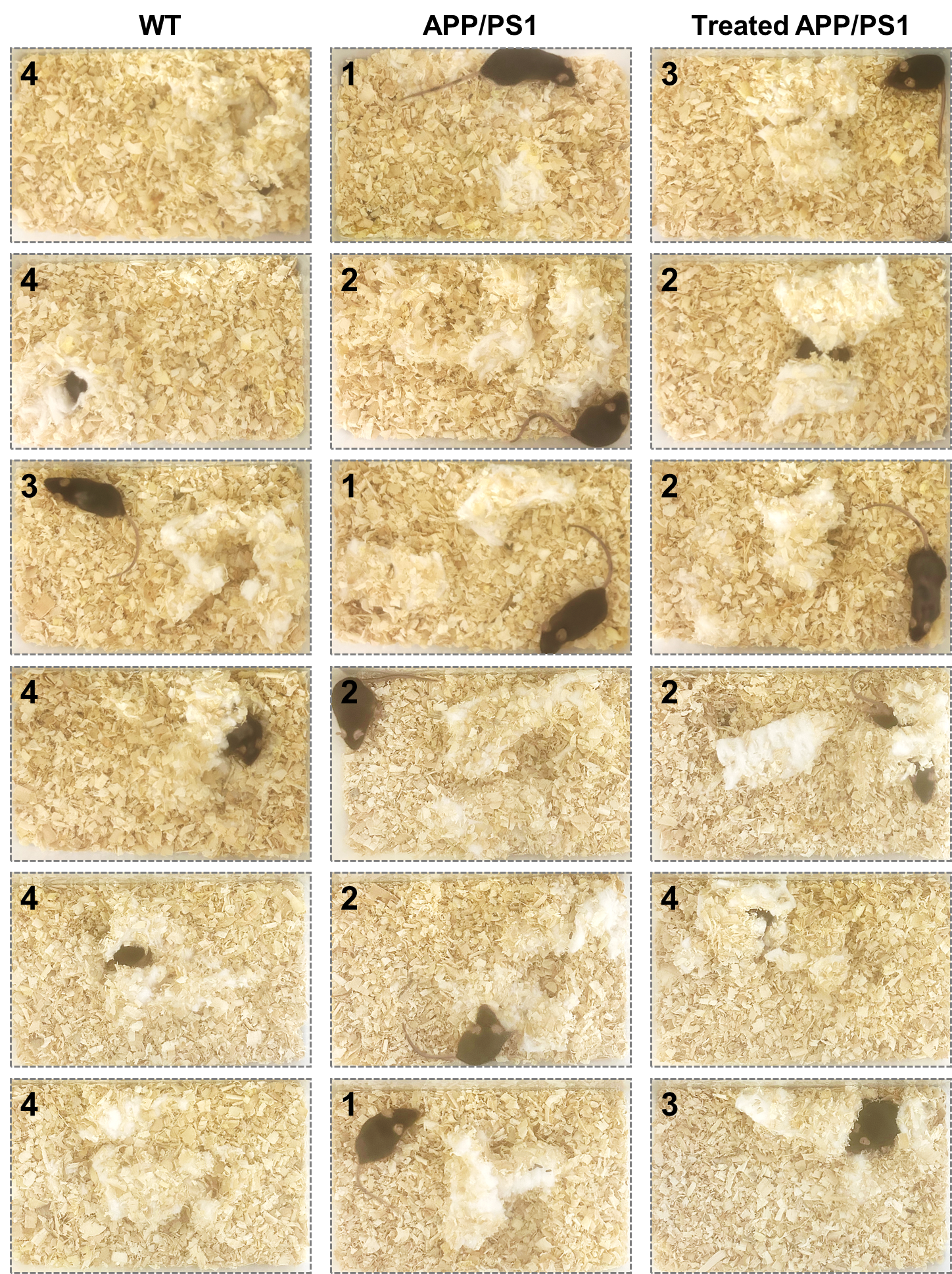
**

**Figure S12. Representative images of mice after the nesting test.** APP/PS1 mice (female, 7-month, n=6) were used in the nesting test. In the nesting test, wood shavings bedding (1.5 cm, 65 g) was added to a newly sterilized mouse cage, and two pieces of cotton were placed side by side at the midpoint of the long side of the cage. Mice were individually placed in the cage, and nesting scores were evaluated after 24 hours according to the following criteria: cotton remains intact with no obvious chewing marks (1 point); cotton are torn into large fragments scattered throughout the cage or piled beneath the mouse to form a shallow nest (2 points); cotton are torn into small fragments and mixed with wood shavings to form a nest with a noticeable gap, or the nest height is below the mouse’s head when the mouse is inside (3 points); cotton are completely torn and mixed with wood shavings to form a nest with no gaps, and the nest height is above the mouse’s head when the mouse is inside (4 points).

**Figure S13. Safety of the SiO_2_@Aβ42 fibril nanovaccines determined by the infiltration of T cells into the mice’s brains.** Brains of the APP/PS1 mice (female, 7-month, n=3) were collected at week 12 after the behavioral testing and the percentages of T cells in live cells in the mice’s brains were measured using flow cytometry.


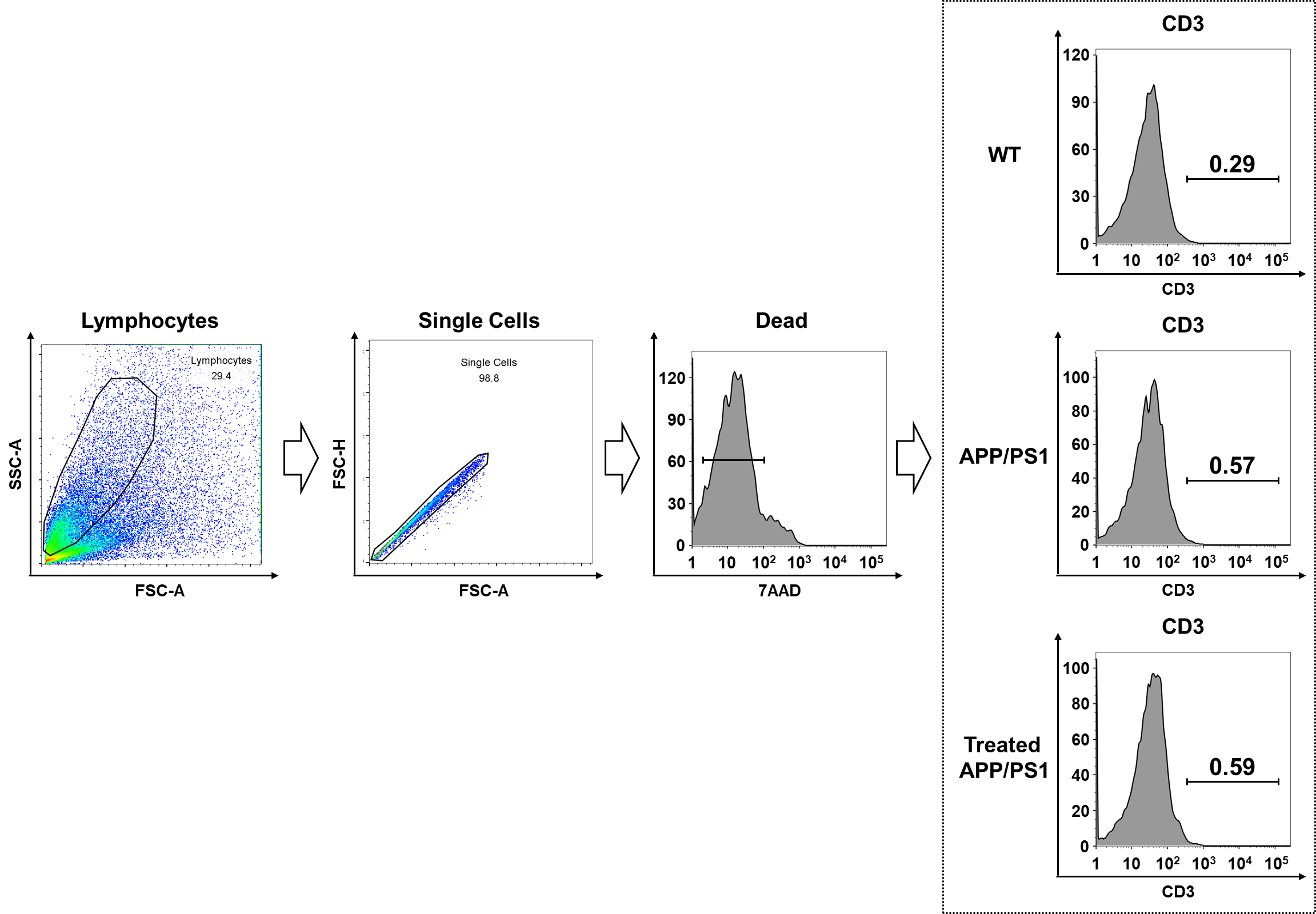


**Figure S14. Percentages of T cells in live cells from the mice’s brains.** Brains were collected from the APP/PS1 mice (female, 7-month, n=4) at week 12 after behavioral testing. Brain cell suspensions were prepared by dissociating the tissues, followed by density gradient centrifugation to isolate brain myelin. The percentages of CD^3+^ cells in live cells were measured using flow cytometry. Values shown in the graphs indicated percentages of positive cells in the representative samples.

**
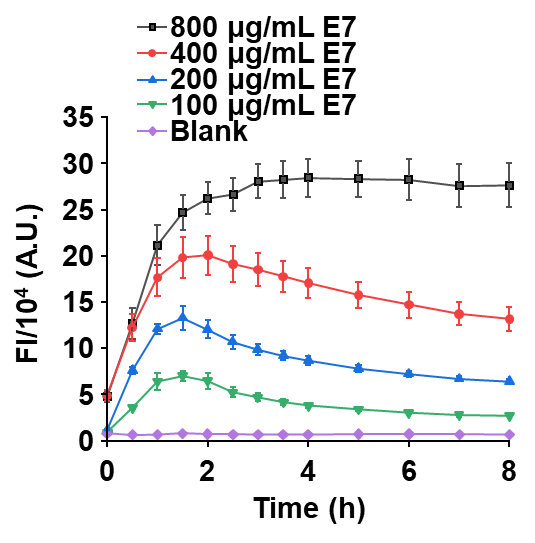
**

**Figure S15. The fibrillation kinetics of the E7 peptide at different concentrations were determined by Thioflavin T (ThT) fluorescence analysis.** The fibrillation kinetics of the E7 peptides were determined using a Thioflavin T (ThT) fluorescence analysis. E7 peptide at concentrations of 100, 200, 400, and 800 μg/mL was incubated in 10 mM HEPES buffer at 300 rpm and 37 °C. The fluorescence intensity of E7 was measured at an excitation (E_x_) wavelength of 440 nm and an emission (E_m_) wavelength of 485 nm.

**
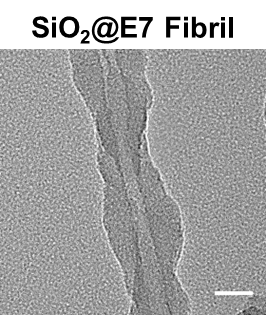
**

**Figure S16.** High resolution TEM images of the SiO_2_@E7 fibril nanovaccines. The scale bar is 20 nm.


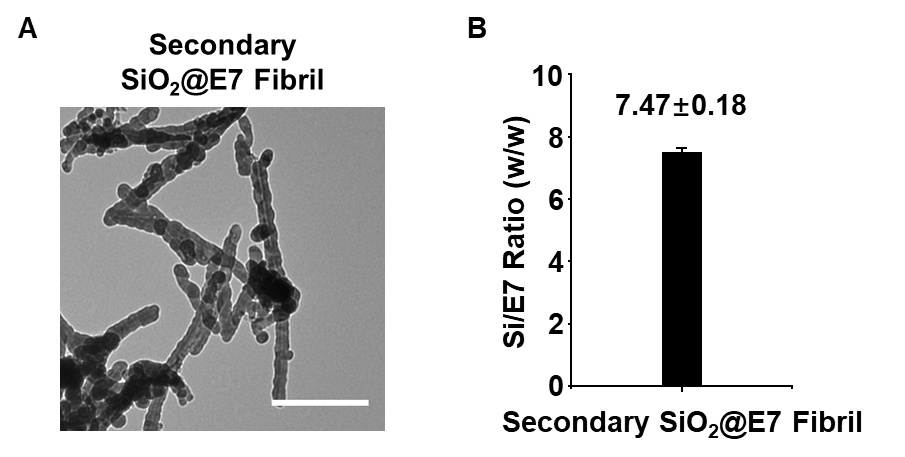


**Figure S17. Characterization of the SiO_2_@E7 fibril nanovaccines prepared by the secondary nucleation-growth strategy.** (A) TEM images of the secondary SiO_2_@ E7 fibril nanovaccines. The scale bar is 500 nm. (B) ICP-OES analysis of the mass ratios of Si to E7 in the secondary SiO_2_@ E7 fibril nanovaccines. n=3.

**Figure S18. Biocompatibility of SiO_2_@E7 fibril nanovaccines**. Cell viability of BMDCs stimulated with SiO_2_@E7 fibril nanovaccines at concentrations of 50 and 100 μg/mL. Cell viability was measured using an MTS assay kit.

**
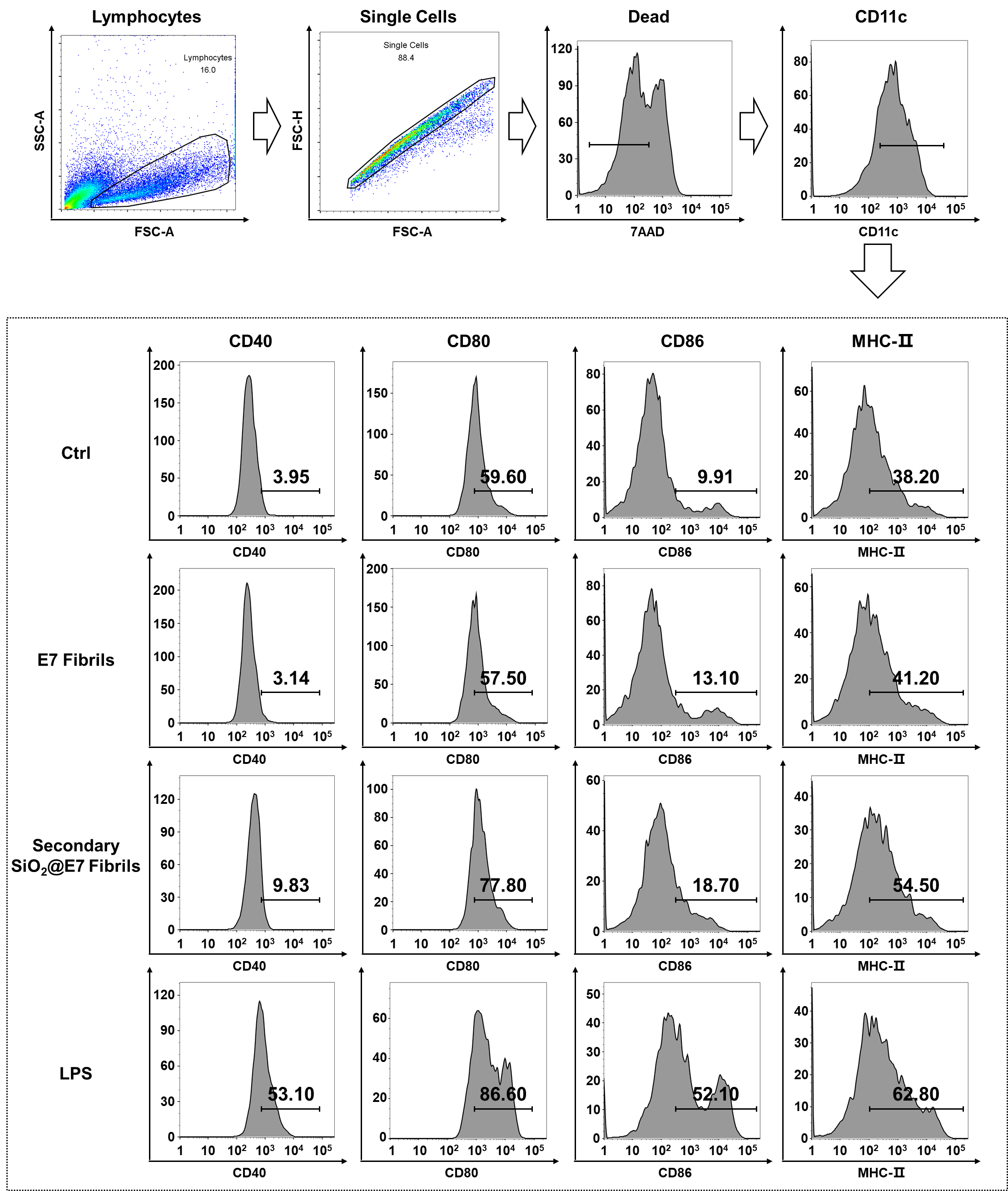
**

**Figure S19. Maturation and activation of BMDCs.** BMDCs were stimulated with SiO_2_@E7 fibril nanovaccines at 100 μg/mL for 24 hours. Cells were then collected, and expression of the surface markers CD40, CD80, CD86, and MHC-II on BMDCs was measured using flow cytometry. Monoclonal antibodies (mAbs) used in the experiment included CD16/CD32, Anti-CD11c Brilliant Violet™ 605, Anti-CD40 Super Bright™ 436, Anti-CD80 FITC, Anti-CD86 APCs, and Anti-I-A/I-E PE. Cell culture medium-treated and E7 fibril-treated BMDCs served as control groups. n=3. Values shown in the graphs indicated percentages of positive cells in the representative samples.

**
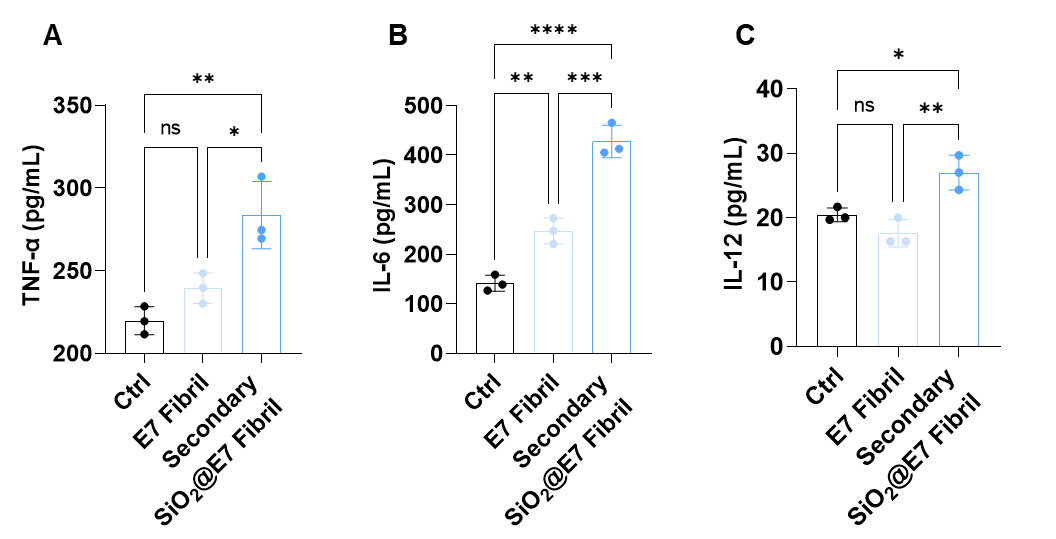
**

**Figure S20. Maturation and activation of BMDCs stimulated with the SiO_2_@E7 fibril nanovaccines.** (A-C) The cytokines TNF-α, IL-6, and IL-12 released from BMDCs induced by the nanovaccines. BMDCs in these experiments were stimulated with the nanovaccines for 24 hours at a concentration of 100 μg/mL. The culture supernatants were then collected, and the concentrations of these cytokines were measured using ELISA. Cell culture medium-treated and E7 fibril-treated BMDCs served as control groups. n=3.


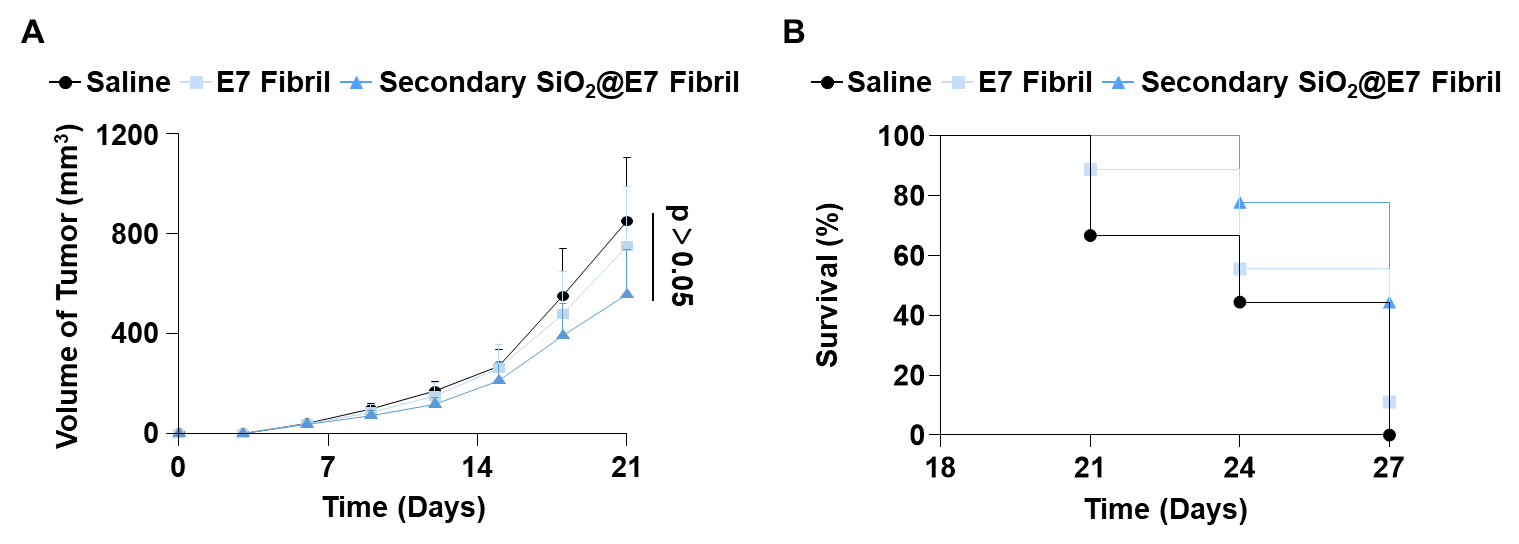


**Figure S21.** **Optimization of the vaccination regimen for SiO_2_@E7 fibril nanovaccines.** (A) Tumor volume (0.5×shortest diameter^2^×longest diameter) growth in mice after 2 immunizations with SiO_2_@E7 fibril nanovaccines. (B) Survival rates of mice after 2 immunizations with SiO_2_@E7 fibril nanovaccines.

**
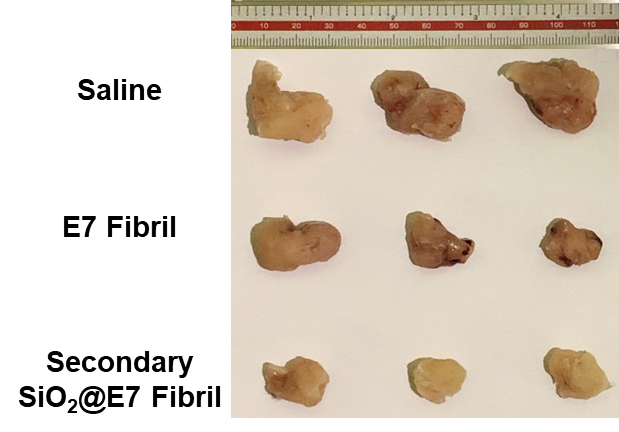
**

**Figure S22. Representative images of tumors in each group.** C57BL/6 mice (female, 6-week, n=8) were used in the experiment. 2×10^5^ TC-1 cells were injected subcutaneously into the right flank of the mice. 5 days after tumor cell inoculation, SiO_2_@E7 fibril nanovaccine containing 10 μg E7 was administered intramuscularly three times at one-week intervals. All mice were sacrificed two weeks after the final immunization. E7 fibril-injected and saline-injected mice served as controls.

**
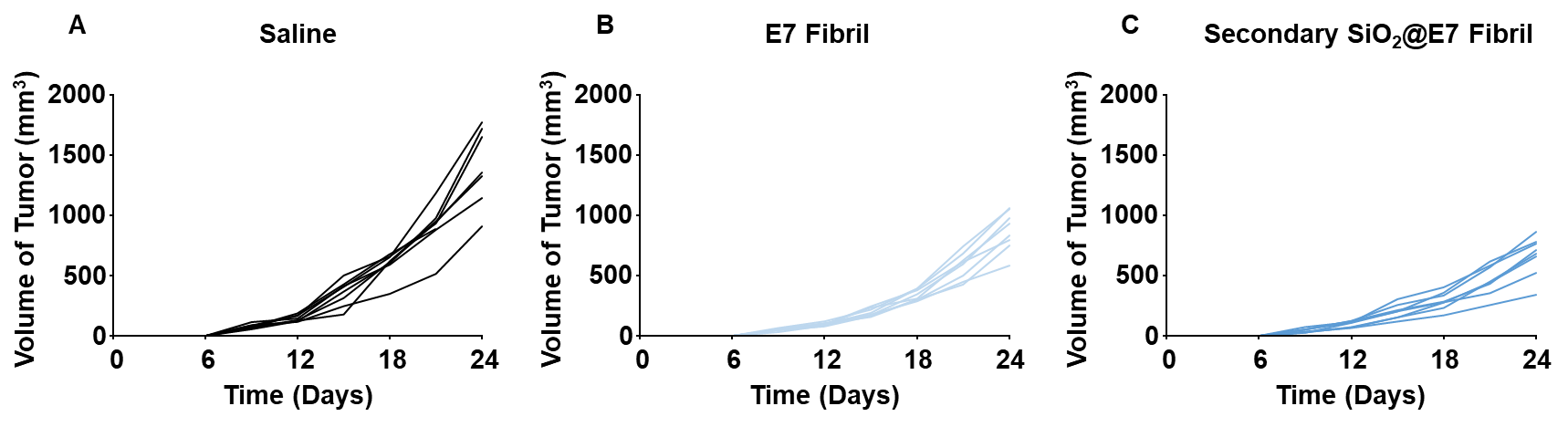
**

**Figure S23. Growth kinetics of tumor volumes for each mouse in each group.** C57BL/6 mice (female, 6-week, n=8) were used in the experiment. 2×10^5^ TC-1 cells were subcutaneously injected into the right flank of the mice. 5 days after tumor cell inoculation, SiO_2_@E7 fibril nanovaccine containing 10 μg E7 was intramuscularly administered three times at one-week intervals. Tumor volume (0.5×shortest diameter^2^×longest diameter) was monitored (mice were sacrificed when the tumor volume >2000 mm^3^). All mice were sacrificed two weeks after the final immunization. E7 fibril-injected and saline-injected mice served as controls.

**
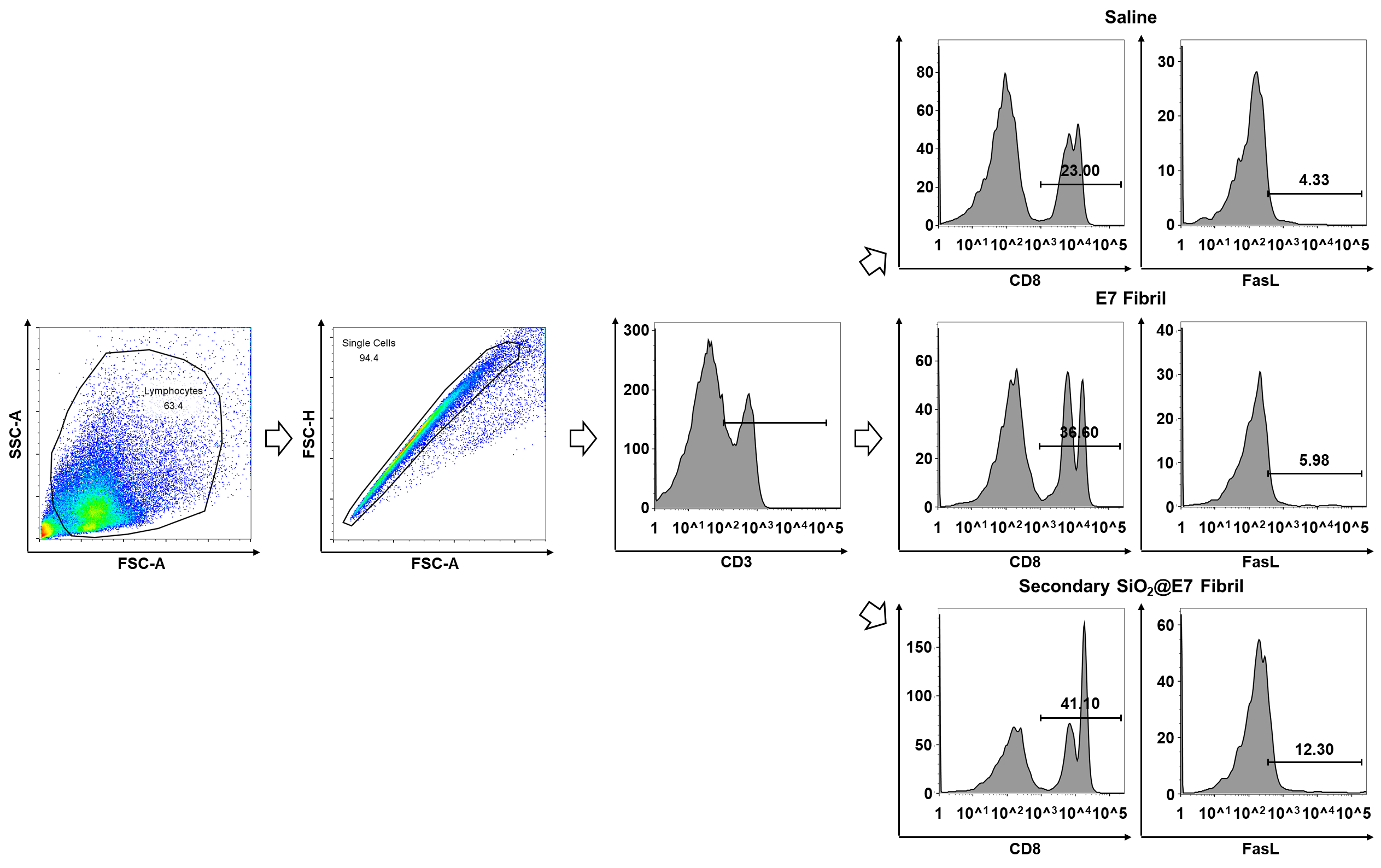
**

**Figure S24. The percentages of CD8^+^ cells in CD3^+^ cells and FsaL^+^ cells in CD8^+^ cells in the spleens determined using flow cytometry.** C57BL/6 mice (female, 6-week, n=8) were used in the experiment. 2×10^5^ TC-1 cells were subcutaneously injected into the right flank of the mice. 5 days after tumor cell inoculation, SiO_2_@E7 fibril nanovaccine containing 10 μg E7 was intramuscularly administered three times at one-week intervals. Spleens isolated from mice were dissociated to prepare cell suspensions, and splenocytes were restimulated with the E7 peptide antigen at 2 μg/mL. The expression of CD8^+^ cells in CD3^+^ cells and the expression of FasL^+^ cells in CD8^+^ cells was determined using flow cytometry. The mAbs used in the experiment included CD16/CD32, Anti-CD3 APC/Cy7, Anti-CD8 APC, and Anti-FasL PE. n=4. Values shown in the graphs indicated percentages of positive cells in the representative samples.

**Table S1. Hydrodynamic sizes, ζ potentials, and average widths of the Aβ42 fibrils, SiO_2_@Aβ42 fibril nanovaccines, and secondary SiO_2_@Aβ42 fibril nanovaccines.**

| **Sample ID** | **Particle Size (nm)** | **PDI** | **Zeta Potential (mV)** | **Width (nm)** |
| --- | --- | --- | --- | --- |
| Aβ42 Fibril | 530±246 | 0.32±0.11 | -34±1 | 10±2 |
| SiO_2_@Aβ42 Fibril | 1330±135 | 0.46±0.01 | 33±1 | 23±3 |
| Secondary SiO_2_@Aβ42 Fibril | 3728±162 | 0.13±0.06 | -33±1 | 42±6 |

**Table S2. Hydrodynamic sizes, ζ potentials, and average widths of the E7 fibrils, SiO_2_@E7 fibril nanovaccines, and secondary SiO_2_@E7 fibril nanovaccines.**

| **Sample ID** | **Particle Size (nm)** | **PDI** | **Zeta Potential (mV)** | **Width (nm)** |
| --- | --- | --- | --- | --- |
| E7 Fibril | 1274±69 | 0.36±0.03 | 5±1 | 11±2 |
| SiO_2_@E7 Fibril | 595±24 | 0.33±0.02 | 31±1 | 19±4 |
| Secondary SiO_2_@E7 Fibril | 3429±758 | 0.52±0.04 | 21±1 | 92±14 |
